# Regulation of chromatin accessibility by the histone chaperone CAF-1 sustains lineage fidelity

**DOI:** 10.1038/s41467-022-29730-6

**Published:** 2022-04-29

**Authors:** Reuben Franklin, Yiming Guo, Shiyang He, Meijuan Chen, Fei Ji, Xinyue Zhou, David Frankhouser, Brian T. Do, Carmen Chiem, Mihyun Jang, M. Andres Blanco, Matthew G. Vander Heiden, Russell C. Rockne, Maria Ninova, David B. Sykes, Konrad Hochedlinger, Rui Lu, Ruslan I. Sadreyev, Jernej Murn, Andrew Volk, Sihem Cheloufi

**Affiliations:** 1 Department of Biochemistry, University of California, Riverside, 3401 Watkins Drive, Boyce Hall, Riverside, CA 92521 United States; 2 Stem Cell Center, University of California, Riverside, 900 University Ave, Riverside, CA 92521 United States; 3 Department of Molecular Biology, Massachusetts General Hospital, 185 Cambridge Street, Boston, MA 02114 United States; 4 Division of Hematology/Oncology, O’Neal Comprehensive Cancer Center, University of Alabama at Birmingham, Birmingham, AL United States; 5 Department of Population Sciences City of Hope National Medical Center, Duarte, CA United States; 6 Koch Institute for Integrative Cancer Research, Massachusetts Institute of Technology, Cambridge, MA 02139 United States; 7 Department of Biology, Massachusetts Institute of Technology, Cambridge, MA 02139 United States; 8 Harvard-MIT Division of Health Sciences and Technology, Harvard Medical School, Boston, MA 02115 United States; 9 Department of Computational and Quantitative Medicine, Division of Mathematical Oncology, City of Hope National Medical Center, Duarte, CA United States; 10 Department of Biomedical Sciences, School of Veterinary Medicine, University of Pennsylvania, Philadelphia, PA United States; 11 Center for Regenerative Medicine, Massachusetts General Hospital, 185 Cambridge Street, Boston, MA 02114 United States; 12 Department of Genetics, Harvard Medical School, 25 Shattuck Street, Boston, MA 02115 United States; 13 Harvard Stem Cell Institute, 1350 Massachusetts Avenue, Cambridge, MA 02138 United States; 14 Cancer Center, Massachusetts General Hospital, 185 Cambridge Street, Boston, MA 02114 United States; 15 Department of Pathology, Massachusetts General Hospital and Harvard Medical School, Boston, MA United States; 16 Experimental Hematology and Cancer Biology, Cincinnati Children’s Hospital Medical Center, Cincinnati, OH United States

**Keywords:** Gene silencing, Stem-cell differentiation, Haematopoietic stem cells, Chromatin remodelling

## Abstract

Cell fate commitment is driven by dynamic changes in chromatin architecture and activity of lineage-specific transcription factors (TFs). The chromatin assembly factor-1 (CAF-1) is a histone chaperone that regulates chromatin architecture by facilitating nucleosome assembly during DNA replication. Accumulating evidence supports a substantial role of CAF-1 in cell fate maintenance, but the mechanisms by which CAF-1 restricts lineage choice remain poorly understood. Here, we investigate how CAF-1 influences chromatin dynamics and TF activity during lineage differentiation. We show that CAF-1 suppression triggers rapid differentiation of myeloid stem and progenitor cells into a mixed lineage state. We find that CAF-1 sustains lineage fidelity by controlling chromatin accessibility at specific loci, and limiting the binding of ELF1 TF at newly-accessible diverging regulatory elements. Together, our findings decipher key traits of chromatin accessibility that sustain lineage integrity and point to a powerful strategy for dissecting transcriptional circuits central to cell fate commitment.

## Abstract (web summary)

Cell fate commitment involves transcription factor activity and changes in chromatin architecture. Here the authors show that CAF-1 maintains lineage fidelity by controlling chromatin accessibility at specific sites; suppressing CAF-1 triggers differentiation of myeloid stem and progenitor cells into a mixed lineage state.

## Introduction

Hematopoiesis involves the sequential commitment of self-renewing hematopoietic stem cells to fully mature specialized blood cell types^1^. During this process, cells become progressively more restricted towards myeloid or lymphoid lineages by a stepwise transition through progenitor cell states. Recent single-cell transcriptome analyses of bone marrow suggest that lineage commitment is heterogeneous and deviates from the largely cell surface marker-based, hierarchical differentiation model of hematopoiesis^1–7^. This signifies the need to understand the molecular mechanisms that sustain the identity of stem and progenitor cells and restrict their commitment to a specific lineage during differentiation.

Lineage specification during hematopoiesis is tightly controlled by transcription factors (TFs). In the myeloid lineage, the CCAAT/enhancer-binding protein (C/EBP) family members play major roles in commitment toward granulocytes and macrophages, while GATA1, KLF1, and GFI1B have been described to govern erythrocyte and megakaryocyte lineage commitment^7^. Notably, some of these TFs, such as CEBPΑ and GATA1, are, upon ectopic expression, sufficient to drive transdifferentiation into a different blood cell lineage^8,9^. However, the mechanisms that sustain hematopoietic lineage integrity remain poorly defined.

During the dynamic process of cellular differentiation, chromatin remodeling typically precedes transcriptional regulation^10^. In this manner, altered chromatin accessibility is thought to set the stage for the activity of key TFs that in turn drive cell lineage specification. Of the many types of molecules implicated in the control of chromatin accessibility, histone chaperones act broadly by catalyzing nucleosome assembly during DNA replication, transcription, and DNA repair^11^. Multiple studies have linked histone chaperones to changes in cell identity, including the replication-dependent chaperone CAF-1, the replication-independent chaperones HIRA and DAXX, and the transcriptionally related chaperones FACT and SPT6^12,13^.

We previously identified the two subunits of the CAF-1 complex, Chaf1a and Chaf1b, as key regulators of cell identity maintenance in different models of transcription factor-driven cellular reprogramming and direct lineage conversions^14^. CAF-1 is a histone chaperone that assembles nucleosomes during DNA replication and is involved in regulating heterochromatin^12,15^. For instance, during cellular reprogramming to pluripotency, CAF-1 blocks the binding of ectopically expressed SOX2 transcription factor by maintaining a closed chromatin state at its target loci. Consistent with this observation, loss of CAF-1 enhances transcription factor-driven reprogramming of somatic cells to pluripotent stem cells and direct lineage conversion of pre-B cells into macrophages and that of fibroblasts into neurons. Given the effect of the CAF-1 complex on chromatin accessibility in these and other cellular paradigms^14–16^, CAF-1 has been viewed as a general stabilizer of cell identity that prevents cells from adopting an open chromatin state characteristic of immature cells. However, studies of CAF-1 function in normal cell and tissue homeostasis have been difficult due to its requirement during DNA replication and hence its essential role in cell proliferation and organismal development^12,15,17,18^.

Recent studies of embryonic stem cell (ESC) differentiation and T cell development uncovered additional roles of CAF-1 in cooperating with chromatin-modifying enzymes, such as the Polycomb repressive complex 2 (PRC2), histone deacetylases (HDAC1/2), the histone demethylase LSD1 and DNA methyltransferases^19–21^. How these activities of CAF-1 might be coupled to the action of TFs and hematopoietic lineage integrity remains to be determined.

Here, we combine a controllable myeloid differentiation system with inducible gene perturbation to investigate the role of CAF-1 in sustaining myeloid stem and progenitor cells and identify the early transcriptional events that control blood lineage fidelity. In this study we demonstrate that CAF-1 deficient stem and progenitor cells display multiple signatures of differentiation along with local chromatin changes that drive heterogeneous transcriptional programs, resulting in a mixed-lineage state. In particular, we show that CAF-1 loss leads to aberrant binding of the ETS transcription factor ELF1 to newly accessible diverging promoter and enhancer elements. Together, our findings uncover how the regulation of chromatin accessibility sustains lineage fidelity and point to a powerful strategy for dissecting transcriptional circuits central to cell fate commitment.

## Results

### CAF-1 maintains the myeloid stem and progenitor cell state

**Fig. 1:**
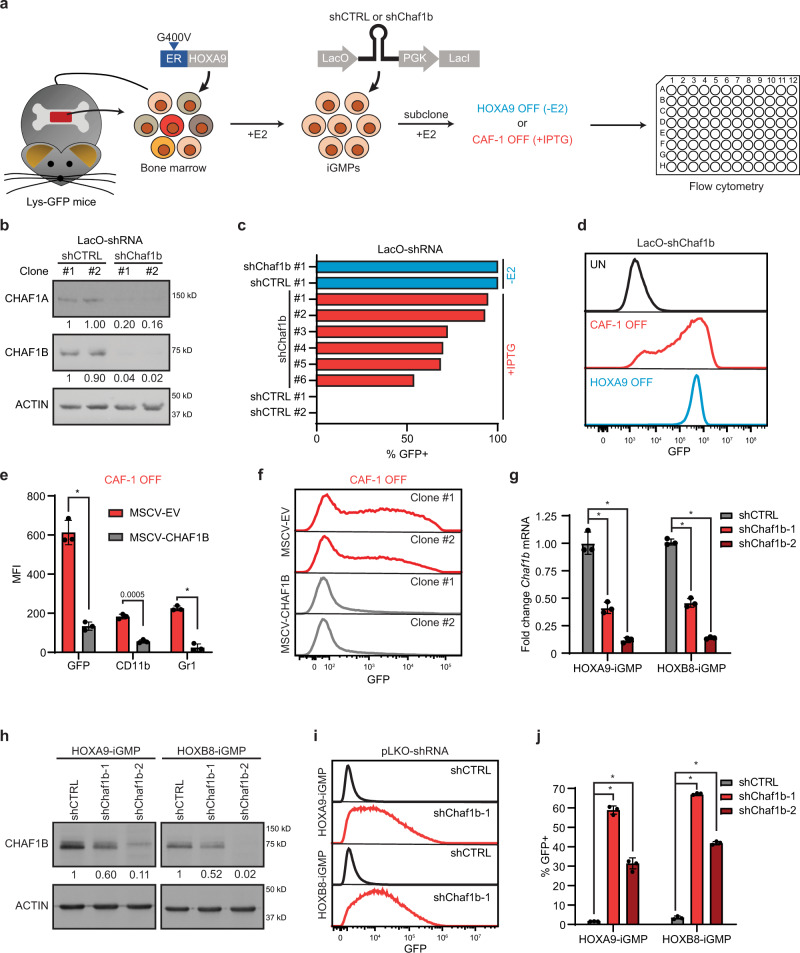
CAF-1 maintains the myeloid stem and progenitor cell state. **a** Derivation of Chaf1b-inducible iGMPs from Lys-GFP bone marrow cells by transducing the HOXA9 TF fused to the ligand-binding domain of the G400V mutant estrogen receptor^22,24^ and IPTG-inducible shRNA lentiviral vectors (LacO-shRNA), expressing either an shRNA targeting Chaf1b (shChaf1b) or a spacer control sequence (shCTRL). Withdrawal of estradiol (−E2) allows for deactivation of HOXA9 (HOXA9 OFF) and IPTG treatment of shChaf1b expressing iGMPs results in CAF-1 depletion (CAF-1 OFF). Cells are analyzed for differentiation 4 days post treatment. **b** Western blot analysis of two independent subclones expressing either shChaf1b or shCTRL treated with IPTG. Two independent experiments were performed with all four subclones with similar results, but one representative experiment is shown. **c** Proportion of Lysozyme-GFP-positive cells (%GFP+) for shChaf1b and shCTRL clones under +IPTG or −E2 treatments. **d** Flow cytometric analysis of GFP signal for shChaf1b clone #1 shown in **c** under uninduced (UN), CAF-1 OFF (+IPTG) or HOXA9 OFF (−E2) conditions. **e** Flow cytometric analysis of three neutrophil markers (GFP, CD11b, and Gr1) in CAF-1 OFF cells overexpressing retroviral vectors with empty control (MSCV-EV) or shRNA-resistant CHAF1B (MSCV-CHAF1B). Mean and standard deviation of mean fluorescence intensity (MFI) is shown for *n* = 3 independent experiments. Two-way ANOVA with Sidak’s correction. **f** Flow cytometric analysis of GFP signal for the rescue experiment as in **e** for two independent subclones. **g**, **h** RT-qPCR and western blot analysis in HOXA9 and HOXB8 iGMP cell lines transduced with lentiviral vectors constitutively expressing shRNAs targeting *Chaf1b* (shChaf1b-1 or shChaf1b-2) or a luciferase shRNA control (shCTRL). **g** The mean and standard deviation of *Chaf1b* mRNA fold change expression of *n* = 3 independent experiments. **h** Representative western blot of three independent experiments with similar results. **i**–**j** Flow cytometric analysis of HOXA9 and HOXB8 iGMPs transduced with shChaf1b-1, shChaf1b-2, or shCTRL. Representative GFP signal is shown in **i** with the mean and standard deviation of % GFP+ population for *n* = 3 independent experiments shown in **j**. Two-way ANOVA with Dunnett’s test. *p < 0.0001. Source data are provided as a Source Data file.

To study the role of CAF-1 in maintaining lineage integrity, we took advantage of a well-established myeloid differentiation system where transient granulocyte-macrophage progenitor (GMP) cells can be captured ex-vivo under self-renewing conditions and induced to differentiate into neutrophils^22,23^. In this system, mouse primary bone marrow cells are transduced with the ligand-binding domain of the G400V mutant estrogen receptor fused to the HOXA9 transcription factor (Fig. 1a). The resulting HOXA9-immortalized GMPs (iGMPs) grown under estradiol (E2) and stem cell factor (SCF) self-renewing culture conditions retain the potential to differentiate into granulocytes and macrophages and remain genetically stable^22,23^. Moreover, these iGMPs also contain a Lysozyme promoter-driven GFP (Lys-GFP) reporter cassette to facilitate monitoring of their normal differentiation into neutrophils^22,24^ upon estradiol (E2) withdrawal (HOXA9 OFF condition; Fig. 1a).

To investigate a putative requirement for CAF-1 in sustaining self-renewal of iGMPs, we established clonal lines expressing an IPTG-inducible shRNA targeting *Chaf1b*, a subunit of the CAF-1 complex (Fig. 1a). iGMP Clones of inducible Chaf1b shRNA were treated with IPTG under self-renewing conditions to activate the shRNA (CAF-1 OFF condition; Fig. 1a). We confirmed a highly efficient knockdown of the CHAF1B protein by comparing to clones infected with an inducible shRNA control vector (Fig. 1b). Notably, CHAF1B depletion also led to degradation of CHAF1A, in agreement with previous observations^14^. We then screened Chaf1b shRNA and control clones for differentiation by monitoring the expression of Lys-GFP (Fig. 1a). Strikingly, after four days of treatment with IPTG, all examined clones bearing the Chaf1b shRNA but not the control vector showed robust induction of GFP, akin to the induction seen during normal differentiation triggered by estradiol withdrawal (Fig. 1c, d). Notably, depletion of CAF-1 subunits had no effect on the expression level of conditional ER-HOXA9, suggesting that CAF-1 OFF cells override the differentiation block sustained by HOXA9 in iGMPs (Supplementary Fig. 1a, b). We also ruled out a potential off-target effect of the shRNA by add-back of an shRNA-resistant CHAF1B (Supplementary Fig. 1c, d), which markedly reduced the expression of GFP and additional myeloid differentiation markers CD11b and Gr1, all of which showed high induction in the CAF-1 OFF state (Fig. 1e, f). In addition, overexpression of CHAF1B alone had little effect on differentiation kinetics of HOXA9 OFF cells (Supplementary Fig. 1e). To further confirm the specificity of the observed phenotypes, we tested two *Chaf1b*-targeting shRNAs in an iGMP cell line that we independently derived using the above ER-HOXA9 fusion transgene (Fig. 1a), and in a separate line generated through immortalization of GMPs using a related ER-HOXB8 fusion transgene. Akin to HOXA9, HOXB8 is a homeobox domain transcription factor that can enforce self-renewal of GMPs ex-vivo and allows for their normal differentiation upon its inactivation^23^. As above, we observed similar differentiation phenotypes upon depletion of CAF-1 with either shRNA in both HOXA9 and HOXB8 derived iGMPs (Fig. 1g–j). Taken together, our results indicate that CAF-1 depletion allows iGMPs to overcome their differentiation blockade, suggesting that this histone chaperone complex maintains the progenitor state of GMPs.

### CAF-1 prevents rapid and partial differentiation of iGMPs

**Fig. 2:**
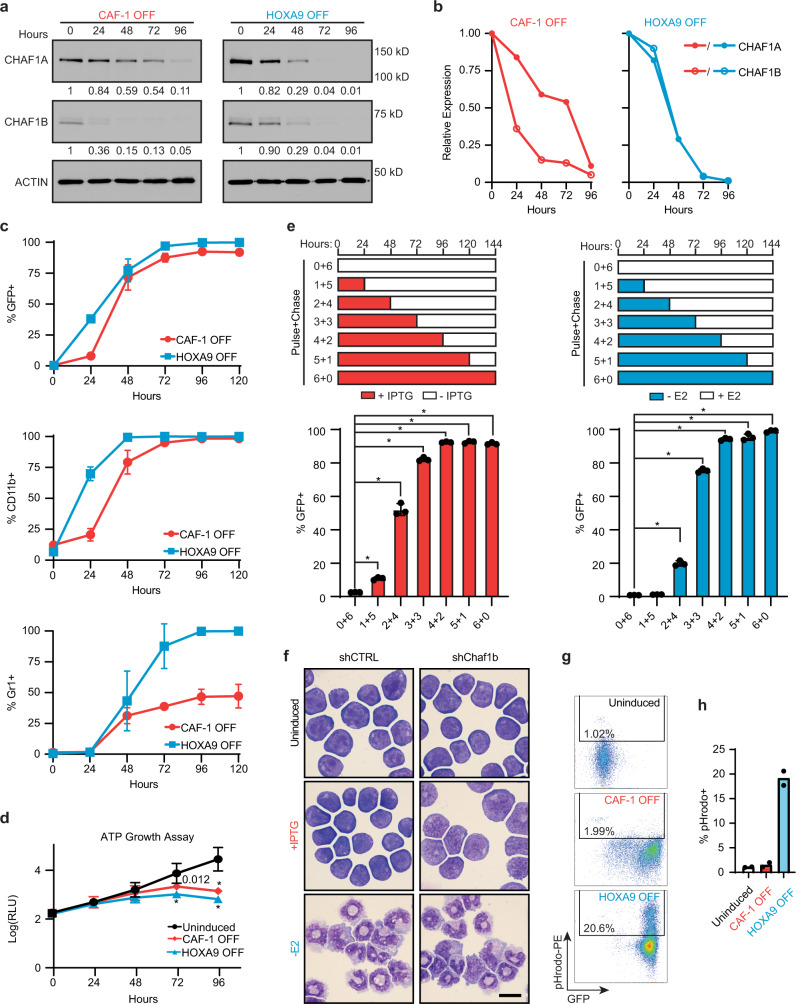
CAF-1 depletion induces rapid and partial differentiation of iGMPs. **a** Western blot time-course analysis of CHAF1A and CHAF1B in CAF-1 OFF and HOXA9 OFF conditions showing a closely matching downregulation trend of both CAF-1 subunits. Two independent experiments were performed with similar results, but one representative experiment is shown. **b** Quantification of the western blot time course in **a**. **c** Flow cytometric time-course analysis showing activation of three neutrophil markers (GFP, CD11b, and Gr1) in CAF-1 OFF and HOXA9-OFF conditions. Mean and standard deviation of marker positive percent population is plotted for *n* = 3 experimental replicates. See corresponding representative histograms in Supplementary Fig. 2b. **d** ATP growth assay time course of uninduced, CAF-1 OFF and HOXA9 OFF cells. Mean and standard deviation of log_10_ transformed relative luminescence units (RLU) is plotted for *n* = 3 experimental replicates. Two-way ANOVA with Dunnett’s test. **e** Top: Schematic of cellular differentiation commitment assay in either CAF-1 OFF or HOXA9 OFF conditions. Shown are CAF-1 OFF (+IPTG) or HOXA9 OFF (−E2) pulses of 24 h increments followed by chase periods of no induction. Bottom: Mean and standard deviation of % GFP+ population from *n* = 3 experimental replicates. Each bar represents a “pulse+chase” condition analyzed 6 days post-induction. Two-way ANOVA with Dunnett’s test. *p < 0.0001. **f** Morphological analysis of clones expressing shCTRL or shChaf1b in uninduced or 96 h post-induction of each treatment. Scale bar = 10 µm. Three independent experiments were performed with similar results, but one representative experiment is shown. **g** Phagocytosis flow cytometric assay. Density plots of shChaf1b expressing clone in uninduced and 96 h post-induction of each treatment showing GFP signal versus fluorescently labeled bacterial particles (pHrodo). pHrodo positive cells are gated. **h** Quantification of % pHrodo+ populations shown in **g** for two independent subclones. Two independent experiments were performed with similar results, but one representative experiment is shown. Source data are provided as a Source Data file.

To understand the kinetics and stability of the observed phenotype upon CAF-1 depletion in iGMPs, we compared the differentiation and growth of CAF-1 OFF cells to HOXA9 OFF cells, which undergo normal differentiation to neutrophils (Fig. 1a)^22,23^. A time-course analysis of CAF-1 subunit expression confirmed rapid and stable depletion of CHAF1B followed by downregulation of CHAF1A upon treatment with IPTG (CAF-1 OFF condition; Fig. 2a, b). Interestingly, removal of estradiol (HOXA9 OFF condition) led to a similarly potent downregulation of both CAF-1 subunits, with CHAF1A closely matching and CHAF1B lagging by about 24 h behind the kinetics of the respective proteins in CAF-1 OFF cells (Fig. 2a, b).

To assess differentiation, we performed flow cytometric analysis of the myeloid differentiation markers, Lys-GFP, CD11b, and Gr1, and noticed their induction by about 48 h and plateauing by 96 h of inducing cellular differentiation either by inactivation of HOXA9 or depletion of CAF-1 (Fig. 2c and Supplementary Fig. 2a–d). However, we also observed an overall stronger GFP signal and reduced CD11b and Gr1 induction, hinting at differences in cell fate conversion between these conditions (Fig. 2c and Supplementary Fig. 2a–d).

In light of the intimate relationship between cell proliferation and differentiation^12,25,26^, we next sought to determine how the growth rate of iGMPs might be affected by depletion of either CAF-1 or HOXA9 compared to uninduced cells. By measuring cellular ATP levels over time, we observed the first signs of slowed growth after 72 h post-induction in both cell populations, but with HOXA9 OFF cells showing more severe growth repression compared to CAF-1 OFF cells (Fig. 2d).

Given the canonical role of CAF-1 in histone deposition during the S phase of the cell cycle, and hence its requirement for cell proliferation^27,28^, an argument could be made that the observed differentiation of iGMPs resulted from the proliferative arrest upon CAF-1 depletion. To investigate this possibility, we focused on CAF-1 OFF cells at 48 h of induction with IPTG, when cells begin expressing differentiation markers, and show no apparent change in growth rate as compared to uninduced cells (Fig. 2c, d and Supplementary Fig 2a–d). Analysis of the cell cycle confirmed a largely unaltered proliferative status of CAF-1 OFF cells at 48 h, with a small but significant increase of cells in S phase, contrasting with HOXA9 OFF cells that have already accumulated in G0/G1 phase at this time of induction (Supplementary Fig. 3a, b). Furthermore, the GFP-positive subpopulation of CAF-1 OFF cells showed a comparable cell cycle profile to that of GFP-negative cells, indicating that the differentiating cells have not yet slowed down their cycling rate at 48 h of CAF-1 depletion (Supplementary Fig. 3c–e). In contrast, both GFP-positive and -negative HOXA9 OFF cells have increased their fractions of the G0/G1 population by 48 h of induction, suggesting that attenuated growth rate preceded the onset of differentiation upon HOXA9 inactivation. To further probe the idea that the onset of iGMP differentiation does not result from the proliferative block in CAF-1 OFF cells, we investigated how an early growth arrest might affect differentiation upon CAF-1 depletion or HOXA9 inactivation (Supplementary Fig. 3f–i). To accomplish this, we depleted the SCF cytokine from the growth media, which induced growth arrest in just 12 h^29^ (Supplementary Fig. 3f, g). In the CAF-1 OFF condition, cells were first induced with IPTG for 12 h followed by SCF depletion, ensuring concomitant CAF-1 depletion and growth arrest by 24 h, a time that precedes any notable phenotypic changes (Fig. 2c, Supplementary Fig. 2a–d), while in the HOXA9 OFF condition, estradiol was removed following SCF depletion (Supplementary Fig. 3f). Remarkably, the growth arrest triggered by SCF depletion led to a substantial differentiation block in CAF-1 OFF cells but had little effect on the differentiation of HOXA9 OFF cells (Supplementary Fig. 3h, i). These results demonstrate that growth inhibition does not promote cell differentiation upon CAF-1 depletion but, on the contrary, suggests that cell division is required for differentiation of CAF-1 OFF cells, possibly due to the S-phase coupled activity of CAF-1. This is in stark contrast to normal differentiation where growth arrest did not block differentiation. Taken together, our findings indicate that CAF-1 depletion in GMPs activates a unique differentiation program whose onset does not result from the proliferative arrest in these cells.

To investigate the dynamics of cell commitment to differentiation upon CAF-1 depletion, we performed a pulse-chase experiment to define the ‘point of no return’, i.e., the minimum time of induction necessary for cell populations to stably commit to their altered phenotype. Cells were inducedby the addition of IPTG or withdrawal of E2 for one and up to 5 days (pulse period), and thereafter returned to normal growth media until day 6 (chase period) when they were analyzed for GFP expression by flow cytometry (Fig. 2e). We found that the majority of responsive CAF-1 OFF cells stably committed to differentiation after just 48 h of induction, which is slightly earlier than HOXA9 OFF cells whose majority first committed at 72 h of induction (Fig. 2e).

Finally, given the many phenotypic similarities but also differences between the differentiation kinetics of CAF-1 OFF and HOXA9 OFF cells, we wondered whether the differentiated cells obtained via CAF-1 depletion mimicked terminally differentiated neutrophils in their morphology and phagocytic ability. Curiously, we found that even after extended times of induction, the morphology of CAF-1 OFF cells appeared quite dissimilar to mature neutrophils generated by HOXA9 inactivation (Fig. 2f). Moreover, a phagocytic assay revealed that, unlike HOXA9 OFF cells, CAF-1 depleted cells do not uptake bacterial particles, indicating their functional incompetence (Fig. 2g, h). Together, these results show that CAF-1 depletion does not induce fully mature, functional neutrophils, suggesting a partial or alternative differentiation pathway.

### CAF-1 depletion in iGMPs results in a mixed-lineage state

To gain a deeper understanding of how depletion of CAF-1 might terminate the self-renewal of iGMPs and instruct their differentiation, we first sought to define the expression profile of CAF-1 OFF cells. Given the general role of CAF-1 in chromatin organization and anticipating potentially heterogeneous effects on individual cells, we conducted transcriptome analysis at single-cell resolution (scRNA-seq) to capture a broad spectrum of possible responses to CAF-1 depletion (Supplementary Fig. 4a–c). We carried out the experiment in uninduced iGMPs and at two timepoints post-induction: at 48 h, when cells began to express markers of differentiation (Fig. 2c and Supplementary Fig. 2a–d), have not yet altered their growth rate (Fig. 2d and Supplementary Fig. 3a–e) and first showed commitment to differentiation (Fig. 2e), and at 96 h, when the stably differentiated and growth-arrested cells were observed (Fig. 2c–e).

**Fig. 3:**
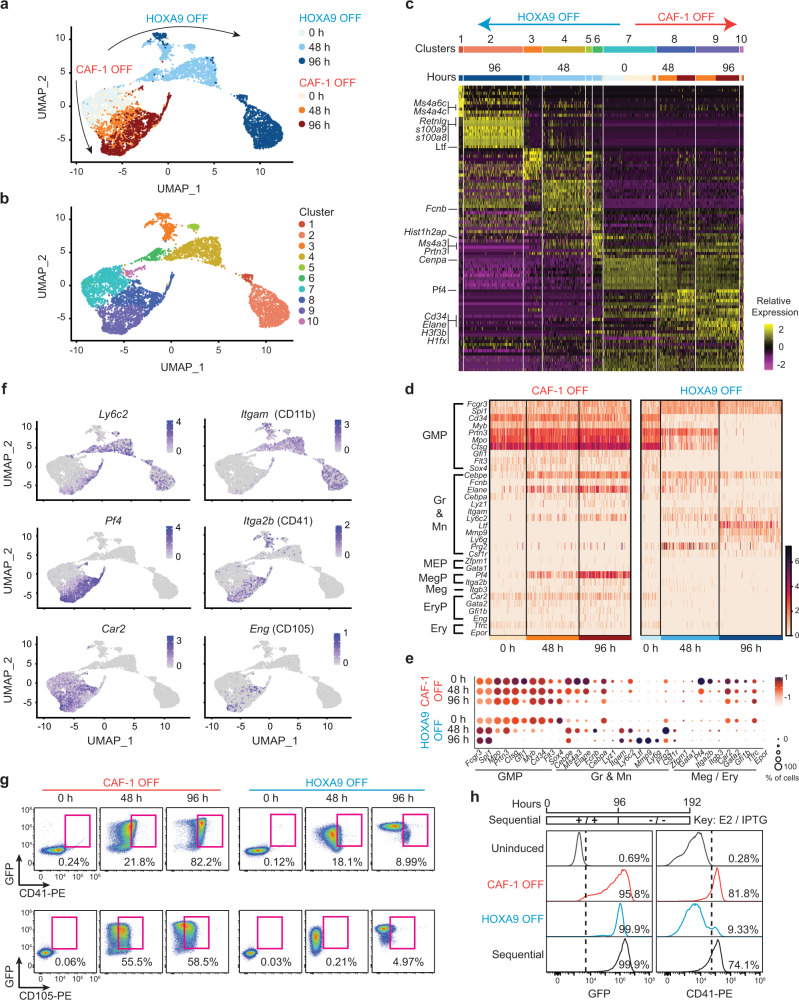
CAF-1 depletion in iGMPs results in a mixed-lineage state. **a**, **b** Time series UMAP analysis of single-cell transcriptomes in iGMPs upon induction of HOXA9 OFF or CAF-1 OFF in shCTRL or shChaf1b expressing clones, respectively. Single cells are colored based on timepoints in **a** or unsupervised clustering in **b**. Clustering was performed with the SLM algorithm using PC1-10 (see methods). **c** Heatmap ordered left to right by clusters 1–10 shown in **b** displaying relative expression of the top 10 enriched genes in each cluster. Corresponding time-course samples are highlighted on the top of the heatmap. Select candidate genes discussed in the text are highlighted to the left of the heatmap. **d** Heatmap of single-cell time series of HOXA9 OFF and CAF-1 OFF inductions displaying relative gene expression levels of a curated set of hematopoietic lineage genes: GMP granulocyte-macrophage progenitor, Gr granulocyte, Mn monocyte/macrophage, MEP megakaryocyte-erythroid progenitor, MegP megakaryocyte progenitor, Meg megakaryocyte, EryP erythroid progenitor, Ery erythrocyte. **e** Bubble plots reflecting the percentage of cells expressing the lineage markers shown in **d** and their abundance in CAF-1 OFF versus HOXA9 OFF cells. The color of the dots shows the relative expression level, and the size of the dots represents the percentage of cells that express the corresponding gene. **f** UMAP feature plots displaying the expression of select markers that characterize different lineages: *Ly6c2* and CD11b for neutrophils, *Pf4* and CD41 for megakaryocyte progenitors and *Car2* and CD105 for erythrocyte progenitors. **g** Time-course flow cytometric analysis of GFP, CD41, and CD105 activation in CAF-1 OFF and HOXA9 OFF cells. Gated populations are cells co-expressing GFP and CD41 or GFP and CD105. **h** Sequential HOXA9 inactivation after CAF-1 depletion. Top panel: schematic for sequential CAF-1 OFF and HOXA9 OFF treatments. Bottom panels: flow cytometric analysis of GFP (left) and CD41(right) in uninduced cells, 96 h post CAF-1 OFF or HOXA9 OFF, and sequential treatment. Fine-tuned sequential and combined pulses from *n* = 3 independent experiments are shown in Supplementary Fig. 5.

Using Uniform Manifold Approximation and Projection (UMAP) analysis^30^, we found diverging cell clusters clearly distinguishing CAF-1 OFF and HOXA9 OFF populations, a trend that became progressively more apparent with increasing time of differentiation (Fig. 3a). We also noticed a maintained similarity between CAF-1 OFF cells and uninduced iGMPs, which contrasted with the distancing differentiation trajectory of HOXA9 OFF cells (Fig. 3a). These findings support our above hypotheses of partial differentiation and induction of an alternate lineage fate.

Fine-resolution unsupervised clustering of differentially expressed genes (DEGs) between clusters identified several gene sets distinguishing the two differentiation trajectories (Fig. 3b, c). For instance, the top genes with relative enrichment in the HOXA9 OFF state included granulocyte maturation markers *s100a8*, *s100a9*, and *Ltf* (Fig. 3c). In contrast, cells in the CAF-1 OFF state exhibited markedly higher expression of genes broadly associated with hematopoietic progenitors (e.g., *Cd34*, *Prtn3*, *Flt3*), histone variant genes (*H3f3b*, *Cenpa*, *Hist1h2ap,* and *H1fx*), and the platelet factor 4 (*Pf4*), a specific marker of megakaryocyte progenitor cells (MegPs). Moreover, several early granulocyte-associated genes, including *Fcnb*, *Elane,* and *Ms4a3*, were only transiently expressed upon HOXA9 inactivation, as expected, but showed sustained upregulation in the CAF-1 OFF condition (Fig. 3c–e). These expression profiles and clustering patterns are consistent with the observation that CAF-1 depletion leads to alternative and incomplete differentiation of myeloid progenitors.

To resolve whether CAF-1 OFF cells are individually locked into a mixed-lineage state or exhibit heterogeneously differentiated populations, we scrutinized a curated set of hematopoietic lineage genes, including cell surface markers and transcription factors (Fig. 3 d, e). As noted above, CAF-1 OFF cells retained strong expression of multiple GMP-associated genes, possibly due to maintained expression of HOXA9 in these cells (Supplementary Fig. 1a, b and see below), suggesting their incomplete exit from the progenitor state (Fig. 3d, e). Interestingly, we found a distinct upregulation of granulocyte and monocyte (Gr and Mn) genes (*Ly6c2*, *Lyz1*, *Cebpe*), erythrocyte progenitor (EryP) markers (*Car2*, *Gata2*, *Eng*/*Cd105*, *Gfi1b*) and MegP markers (*Pf4*, *Itga2b*/*Cd41*) (Fig. 3d–f). Markedly, when we analyzed the subset of single cells expressing the Gr1 neutrophil marker, we found that expression of the lineage-specific markers was not limited to different subsets of CAF-1 OFF cells but that the majority of cells co-expressed several of these genes, indicating a mixed cellular state (Supplementary Fig. 4d). To check whether these transcript-level observations might be manifested on the protein level, we assayed cell surface expression of the MegP marker ITGA2B (CD41) and EryP marker ENG (CD105) in each differentiation condition. Consistent with the scRNA-seq analysis, we found co-expression of both markers along with GFP uniquely upon CAF-1 suppression (Fig. 3g). The mixed-lineage state of CAF-1 OFF cells was reproducibly observed in an independent iGMP subclone, which further pointed to a correlation between CAF-1 dosage and the strength of induction of markers from different lineages (Supplementary Fig. 4e, f). Taken together, co-expression of myeloid, erythrocytic, and megakaryocytic lineage-specific genes along with the retention of myeloid progenitor markers upon CAF-1 suppression in GMPs suggests incomplete differentiation and a mixed-identity cellular state.

We next assessed whether the persistent activity of HOXA9 could account for the induction of the diverse lineage markers in CAF-1 OFF cells. To test this directly, we inactivated HOXA9 following, or simultaneously with, CAF-1 depletion at different time intervals (Fig. 3h and Supplementary Fig. 5a). This led to a further increase in the proportion of cells expressing the neutrophil reporter marker Lys-GFP, validating a maintained functionality of the HOXA9-ER inducible transgene (Fig. 3h and Supplementary Fig. 5a, b). Notably, despite inactivation of HOXA9 following, or simultaneously with, depletion of CAF-1, we still detected an increase in the proportion of cells expressing the MegP marker CD41, suggesting that the maintained activity of HOXA9 had no significant impact on the induction of lineage-specific genes in CAF-1 OFF cells (Fig. 3h and Supplementary Fig. 5a, c). These data indicate that loss of CAF-1 sets GMPs on the trajectory of a mixed-lineage state independently of HOXA9 (Fig. 3h and Supplementary Fig. 5a–c and see below).

### CAF-1 loss induces a mixed-lineage program in primary cells

**Fig. 4:**
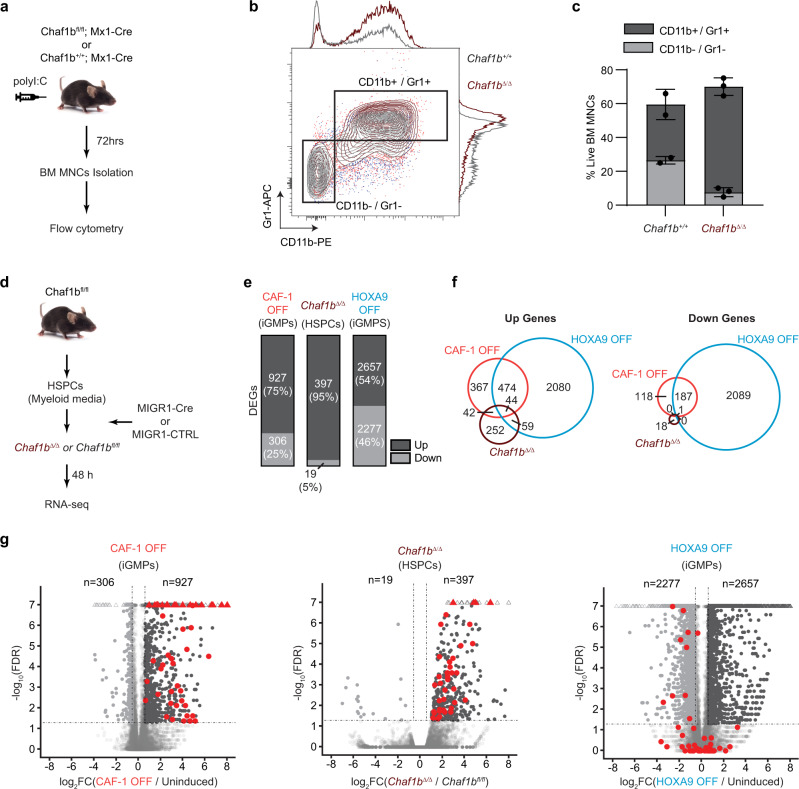
CAF-1 depletion induces myeloid differentiation in vivo and a mixed-lineage transcriptional signature in HSPCs. **a** Schematic of polyI:C (polyinosinic:polycytidylic acid) inducible Mx1-Cre/LoxP system in mice. Flow cytometric analysis of BM MNCs is performed 72 hrs post-induction of mice bearing homozygous *Chaf1b* floxed alleles and Mx1-Cre (*Chaf1b*^*fl*/fl^; Mx1-Cre) and littermate controls (*Chaf1b*^+/+^; Mx1-Cre). **b** Flow cytometric analysis of myeloid differentiation markers (CD11b and Gr1) showing overlaid density plots and histograms of wild-type (Chaf1b ^+/+^) and Chaf1b-deleted (Chaf1b ^Δ/ Δ^) BM MNCs. **c** Quantification of gated populations in **b** showing mean and standard deviation of CD11b and Gr1 double-positive and double negative % populations from live BM MNCs isolated from *n* = 2 wild-type and *n* = 3 knockout littermates. **d** Schematic of *Chaf1b* depletion in primary HSPCs. HSPCs isolated from homozygous floxed mice (*Chaf1b*^*fl*/fl^) are transduced with retroviral vectors, either empty control (MIGR1-CTRL) or overexpressing Cre recombinase (MIGR1-Cre) and analyzed by RNA-seq 48 h following expansion in myeloid media conditions. **e** RNA-seq analysis showing percentages and numbers of upregulated or downregulated genes in CAF-1 OFF iGMPs, HOXA9 OFF iGMPs, and *Chaf1b* ^Δ/ Δ^ HSPCs. DEGs are filtered based on fold change ≥ 1.5 and FDR < 0.05. **f** Venn diagrams of unique and common DEGs between CAF-1 OFF iGMPs, HOXA9 OFF iGMPs, and Chaf1b ^Δ/ Δ^ HSPCs shown in **e**. **g** Volcano plots depicting differential gene expression in CAF-1 OFF iGMPs, *Chaf1b* ^Δ/ Δ^ HSPCs, and HOXA9 OFF iGMPs (Up = dark gray; Down = light gray). Dashed lines demarcate fold change (FC) and FDR cutoffs for DEGs shown in (e-f). −log_10_(FDR) and log_2_(FC) values have been capped at 7 and ±-8, respectively. Triangles (Δ) represent data points exceeding the cap. Genes that are commonly upregulated only in CAF-1 OFF iGMPs and *Chaf1b* ^Δ/ Δ^ HSPCs are shown in red (*n* = 42 genes; see panel **f** and Supplementary Data 1). Source data are provided as a Source Data file.

To determine whether the differentiation induced by CAF-1 depletion in iGMPs could be recapitulated in vivo, we took advantage of the floxed *Chaf1b* Mx1-Cre/LoxP mouse strain that allows for the inducible deletion of *Chaf1b* broadly in hematopoietic cells^18^. Homozygous floxed *Chaf1b* mice carrying a Mx1-Cre knock-in transgene (*Mx1*^*+/−*^*; Chaf1b*^*fl/fl*^) or Mx1-Cre positive wild-type littermate controls (*Mx1*^*+/−*^*; Chaf1b*^*+/+*^) were injected with polyinosine:polycytosine (polyI:C) to induce Mx1-Cre and deplete *Chaf1b* (Fig. 4a). Mononuclear bone marrow cells, which present a mixed population of differentiated, stem, and progenitor hematopoietic cells, were then isolated 72 h post Cre induction and analyzed by flow cytometry for myeloid differentiation markers CD11b and Gr1 (Fig. 4a). The results showed an increased proportion of CD11b/Gr1 double-positive cells in the *Mx1*^*+*^*; Chaf1b*^*fl/fl*^ population (*Chaf1b*^Δ/Δ^), in line with the differentiation phenotype seen upon CAF-1 depletion in iGMPs (Fig. 4b, c and see Fig. 2).

To directly assess parallels with myeloid differentiation of primary cells lacking CHAF1B, we isolated hematopoietic stem and progenitor cells (HSPCs) from the *Chaf1b* floxed mice and induced *Chaf1b* deletion in myeloid culture conditions using retrovirally delivered Cre (Fig. 4d). We then conducted RNA-seq analysis 48 h after delivery of the Cre recombinase (*Chaf1b*^Δ/Δ^) and compared the results with separate bulk population RNA-seq analyses of iGMPs at 48 h of treatment with IPTG (CAF-1 OFF) or withdrawal of estradiol (HOXA9 OFF). Gene Set Enrichment Analysis (GSEA)^31^ revealed similar enrichment of myeloid and HOXA9-regulated genes in all three datasets (Supplementary Fig. 6a). In line with CAF-1’s general repressive effect on gene expression^16,18,21^, we observed a significant bias towards gene upregulation upon CAF-1 depletion in both systems; of all DEGs, 95% and 75% were upregulated in the CAF-1 depleted primary HSPCs and iGMPs, respectively (Fig. 4e). This is in stark contrast to the more balanced upregulated and downregulated genes in the HOXA9 OFF GMPs. Moreover, we detect a marked overlap of upregulated genes with the HOXA9 OFF condition (56% for iGMPs and 26% for primary HSPCs) supporting induction of myeloid differentiation (Fig. 4f). Furthermore, consistent with our scRNA-seq analysis (Fig. 3d–g), the population-wide RNA-seq analysis of CAF-1 OFF iGMPs similarly detected upregulation of genes of diverse blood lineages, including CD105 (*Eng*), *Pf4*, *Gfi1b*, *Tub*(Tubby), and *Cxcl3* (Fig. 4g and Supplementary Data 1). Strikingly, many of these genes were also upregulated in the primary *Chaf1b*-deleted HSPCs, despite their heterogeneous nature and different culture condition, but not in HOXA9 OFF cells, indicating traits of the mixed-lineage differentiation program commonly induced by CAF-1 depletion (Fig. 4g and Supplementary Data 1).

### CAF-1 restricts locus-specific transcription factor binding

**Fig. 5:**
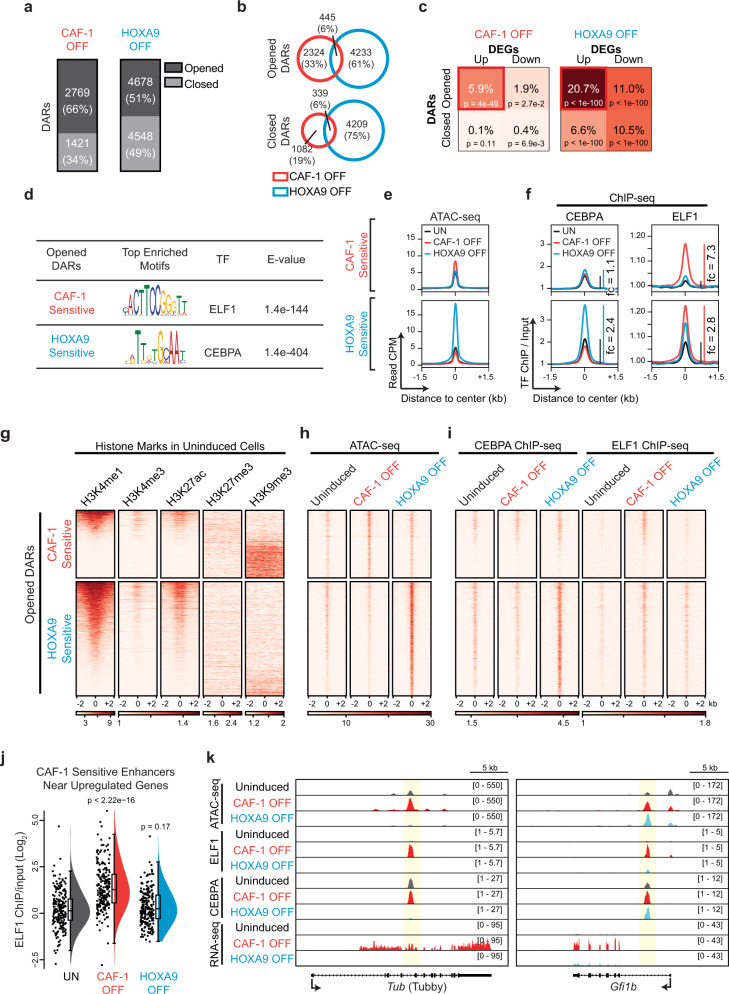
CAF-1 controls chromatin accessibility at specific loci and restricts ectopic binding of ELF1. **a**, **b** Numbers and fractions of all opened and closed differentially accessible regions (DARs; fold change > 2 and FDR < 0.01; see Methods) comparing CAF-1 OFF and HOXA9 OFF conditions 48 h post-induction. **c** Matrix comparing the fractions of opened and closed DARs within 100 kb of upregulated or downregulated genes in CAF-1 OFF and HOXA9 OFF conditions. **d** Top de-novo TF motif predictions in either CAF-1 OFF or HOXA9 OFF opened DARs termed sensitive sites. **e** Metaplots of median ATAC-seq counts per million (CPM) centered at CAF-1 or HOXA9-sensitive sites for all conditions. **f** Metaplots of CEBPA and ELF1 mean ChIP-seq signal over input centered at CAF-1 and HOXA9-sensitive sites for all conditions. Colored vertical lines show TF enrichment fold change (fc) relative to baseline signal in uninduced cells (black) for ELF1 in CAF-1 OFF cells (red) and for CEBPA in HOXA9 OFF cells (blue). **g**–**i** Chromatin profiles of sensitive sites sorted based on H3K4me1 enrichment. **g** histone marks enrichment (ChIP-seq/input or CUT&RUN signal/IgG control) in uninduced cells. **h** ATAC-seq signal (CPM) for all conditions. **i** TF binding (ChIP-seq/input) for all conditions. **j** Raincloud plots showing ELF1 enrichment in all conditions over CAF-1 sensitive enhancers (*n* = 210 sites) within 100 kb of genes upregulated in CAF-1 OFF (204 genes). Boxplots and density curves summarize signal distribution with center lines as the median, box bounds as the interquartile range, and whiskers defining the 95% confidence interval (CI). Welch’s *t*-test for unequal variances (UN vs. CAF-1 OFF/HOXA9 OFF: t = 11.711/1.3657, df = 673.88/415.78, effect size = 1.10/0.133, CI = 95%) UN uninduced. **k** Representative loci harboring sensitive sites that are proximal to genes upregulated upon CAF-1 depletion showing ATAC-seq, TF ChIP-seq, and RNA-seq tracks in all conditions. ATAC-seq and RNA-seq signal is normalized to library size and ChIP-seq signal is normalized to input. Shaded regions highlight sensitive sites with corresponding binding of TFs. Source data are provided as a Source Data file.

We next asked how CAF-1 depletion might result in the mixed-lineage state of cells. Given the canonical role of CAF-1 in chromatin assembly^12,15^ and upregulation of the majority of DEGs upon CAF-1 depletion (Fig. 4e–g), we hypothesized that CAF-1 may act by exerting a direct effect on chromatin accessibility. To investigate this, we employed ATAC-seq to detect changes in chromatin accessibility at different times after induction with IPTG. Whereas only minimal changes were seen at 24 h of treatment, a more substantial effect on chromatin accessibility was apparent at 48 h of induction in the CAF-1 OFF state, consistent with changes to the transcriptome at corresponding times (Supplementary Fig. 6b, c). In addition, consistent with a strong bias toward gene activation seen in the CAF-1 OFF cells (Fig. 4e–g), we observed a similar bias of differentially accessible regions (DARs) toward chromatin opening upon CAF-1 depletion, a trend not seen in the HOXA9-OFF state (Fig. 5a). Furthermore, in line with the disparate differentiation trajectories of two conditions (Fig. 3d-g, Fig. 4e–g and Supplementary Data 1), CAF-1 OFF cells share only a small fraction of DARs with HOXA9 OFF cells; of all DARs, 6.4% and 6% are commonly opened and closed, respectively (Fig. 5b). A closer correlative analysis of the ATAC-seq and RNA-seq data revealed a significant enrichment of opened DARs in the vicinity of upregulated genes (Fig. 5c). Taken together, these observations suggest that altered chromatin accessibility plays a major role in defining the transcriptional profile of iGMPs during their differentiation induced by CAF-1 depletion.

We then asked whether the increase in chromatin opening after CAF-1 depletion may promote the association of diverse lineage-specific TFs with regulatory regions on chromatin to drive the establishment of the observed mixed cellular state. To identify such factors, we looked for enrichment of TF-binding motifs within the opened DARs resulting from depletion of CAF-1 or HOXA9 inactivation, hereafter referred to as “CAF-1 sensitive sites” or “HOXA9 sensitive sites”, respectively (Fig. 5d, e). Multiple Expectation maximizations for Motif Elicitation (MEME)^32^ analysis identified a sequence recognized by the E74 Like ETS transcription factor ELF1 as the top enriched motif in the CAF-1-sensitive sites, whereas CEBPA recognition motif was a dominant enriched consensus sequence in the HOXA9-sensitive sites (Fig. 5d). Notably, in agreement with the observed phenotypes, CEBPA is required for myeloid differentiation^33–35^, while ELF1 is known primarily for its involvement in regulating immune responses and has been proposed to regulate erythroid differentiation as well as the development of natural killer cells and T cells^36,37^. Indeed, previous analyses suggest a broader expression profile for ELF1 during hematopoiesis compared to CEBPA^38^ (Supplementary Fig. 7a, b), pointing to the contribution of ELF1 to the mixed cellular state of CAF-1 OFF cells. Of note, these TFs show no significant changes in expression during the initial 48 h of CAF-1 depletion, arguing that the observed differentiation phenotype is not driven by the induction of either TFs (Supplementary Fig. 7c, d). Together, the correlation of changes in chromatin accessibility and gene expression along with our identification of ELF1 and CEBPA binding motifs suggests that depletion of CAF-1 facilitates chromatin accessibility primarily to ELF1, whereas HOXA9 inactivation promotes chromatin binding of CEBPA leading to activation of target genes.

To probe our motif search predictions, we performed ChIP-seq analyses of CEBPA and ELF1 in iGMPs prior to and at 48 h post-CAF-1 OFF or HOXA9 OFF induction. Consistent with our predictions, we detected a significantly increased binding of CEBPA and a moderately increased binding of ELF1 at HOXA9-sensitive sites, whereas CAF-1-sensitive sites showed an exclusive enrichment in ELF1 but not CEBPA binding (Fig. 5f). To further characterize the nature of the CAF-1-sensitive and HOXA9-sensitive sites identified by ATAC-seq, we performed ChIP-seq or CUT&RUN analyses of active and repressive histone marks in uninduced iGMPs. We found that whereas the CAF-1-sensitive sites segregated into enhancer/promoter regulatory elements (marked by H3K4me1, H3K4me3, and H3K27ac) and heterochromatic regions (marked by H3K9me3), the HOXA9-sensitive sites showed almost exclusively a signature of enhancer/promoter elements (Fig. 5g; Supplementary Fig. 7e). Given that the large majority of CAF-1-sensitive (86%) and HOXA9-sensitive (88%) sites with active histone marks are located more than 2 kb upstream and more than 0.5 kb downstream of the annotated transcription start sites of coding genes (Fig. 5g and see methods), we postulate that the majority of these sites operate as enhancers with the remainder localizing to promoter regions of genes. The restriction of ELF1 and CEBPA binding to promoter/enhancer elements and not the heterochromatic CAF-1 sensitive sites is thus in line with the expected sites of action for TFs (Fig. 5g–i). Importantly, consistent with the correlation of CAF-1-sensitive sites and upregulation of their proximal genes in the CAF-1 OFF condition (Fig. 5c), we detected a significant increase in ELF1 binding at this subset of CAF-1-sensitive enhancer sites, pointing to a direct role of ELF1 in promoting the mixed-lineage state (Fig. 5j). Indeed, we detected increased binding of ELF1 at genic loci that become transcriptionally active when CAF-1 is depleted (Fig. 5k and Supplementary Fig. 8a). Notably, several of these genes mark diverse lineages during hematopoiesis (Supplementary Fig. 8b). Taken together, these analyses identify ELF1 as a candidate regulator of the mixed-lineage cellular fate upon suppression of CAF-1.

### CAF-1 controls lineage-specific transcription factor activity

**Fig. 6:**
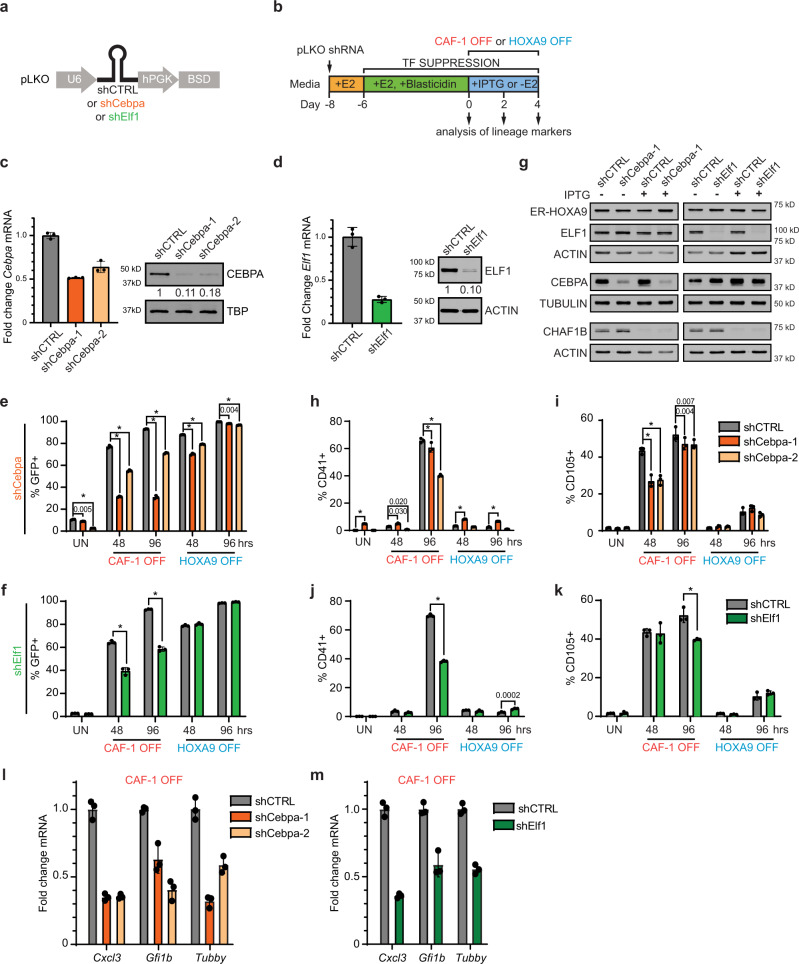
ELF1 and CEBPA are required for the mixed-lineage state induced by CAF-1 depletion. **a** Schematic of a lentiviral vector expressing scrambled shRNA control (shCTRL) or shRNAs targeting CEBPA (shCebpa) or ELF1 (shElf1). **b** Strategy for CEBPA and ELF1 loss of function analysis in all conditions. **c**, **d** RT-qPCR and western blot of CEBPA (**c**) and ELF1 (**d**) in uninduced iGMPs transduced with corresponding shRNA vectors. For RT-qPCR assays, the mean and standard deviation of fold change mRNA expression normalized to actin is shown for three technical replicates. Two independent experiments were performed with similar results, but one representative experiment is shown. **e**, **f** Flow cytometric time-course analysis of Lys-GFP activation upon CEBPA (**e**) and ELF1 (**f**) knockdown in CAF-1 OFF and HOXA9 OFF conditions. The mean and standard deviation of % GFP+ population is plotted for *n* = 3 independent experiments. Two-way ANOVA with Dunnett’s test in **e** and Sidak’s correction in **f**. **g** Western blot for HOXA9, ELF1, CEBPA, and CHAF1B in control (shCTRL) and knockdown (shCebpa or shElf1) in uninduced iGMPs and 48 h post IPTG induction. Two independent experiments were performed with similar results, but one representative experiment is shown. **h**–**k** Flow cytometric time-course analysis of CD41 and CD105 activation upon CEBPA knockdown (**h**, **i**) or ELF1 knockdown (**j**, **k**) in CAF-1 OFF or HOXA9 OFF conditions. The mean and standard deviation of % positive population for each markers is plotted for *n* = 3 independent experiments. Two-way ANOVA with Dunnett’s test in **h**, **j** and Sidak’s correction in **i**, **k**. UN uninduced. *p < 0.0001. **l**, **m** RT-qPCR of three alternative lineage target genes upregulated upon CAF-1 depletion with proximal sensitive sites bound by ELF1 and CEBPA (see genome snapshots in Fig. 5k and Supplementary Fig. 8a) in either control (shCTRL), CEBPA knockdown (**l**) or ELF1 knockdown (**m**) iGMPs 48 h post CAF-1 OFF induction. Mean and standard deviation of mRNA expression fold change normalized to *Gapdh* is plotted for three technical replicates. Two independent experiments were performed with similar results, but one representative experiment is shown. Source data are provided as a Source Data file.

To directly investigate a potential functional requirement of CEBPA and ELF1 for the mixed-lineage state upon depletion of CAF-1, we performed loss-of-function studies in iGMPs using RNAi or CRISPR/Cas9-assisted gene editing followed by flow cytometric analysis and RT-qPCR of key target genes (Fig. 6a-d and Supplementary Fig. 9a–c). We first assayed the induction of the Lys-GFP reporter upon CEBPA or ELF1 targeting. Depletion of CEBPA impaired neutrophil differentiation initiated by either loss of CAF-1 or inactivation of HOXA9 (Fig. 6e and Supplementary Fig. 9d, e). In contrast, loss of ELF1 resulted in reduced Lys-GFP induction specifically in CAF-1 OFF cells, in accord with the enrichment of the ELF1 binding at CAF-1 sensitive sites upon depletion of CAF-1 (Fig. 5f, i; Fig. 6f and Supplementary Fig. 9f, g). Importantly, the knockdown of either CEBPA or ELF1 in iGMPs did not affect the levels of ER-HOXA9 or CHAF1B in uninduced and CAF-1 OFF cells (Fig. 6g). We speculate that the stronger effect of CEBPA knockdown on Lys-GFP repression in the CAF-1 OFF condition compared to the HOXA9 OFF condition could be due to the stronger association of CEBPA with chromatin in HOXA9 OFF cells compared to CAF-1 OFF cells (Fig. 5f, i). These results suggest that whereas both CEBPA and ELF1 are required for differentiation of CAF-1 OFF cells, depletion of CAF-1 directly mobilizes ELF1 but not CEBPA for transcriptional activation of differentiation-linked genes.

Finally, to test the hypothesis that CEBPA and ELF1 might contribute to the mixed cellular state of CAF-1 depleted cells, we determined the effect of their individual suppression on the induction of alternate lineage gene targets. Specifically, knockdown of either ELF1 or CEBPA led to suppression of megakaryocyte and erythrocyte markers, CD41 and CD105, respectively in CAF-1 OFF cells, but had no effect in HOXA9 OFF cells (Fig. 6h–k). Moreover, as expected, loss of ELF1 and CEBPA led to repression of alternate lineage gene targets that were bound by both TFs and activated upon CAF-1 depletion in control cells (Fig. 5k, Supplementary Fig. 8a and Fig. 6l, m).

Taken together, we conclude that in GMPs, CAF-1 restricts chromatin accessibility to lineage-specific TFs, in particular ELF1, to prevent spurious cell differentiation and to sustain lineage fidelity (see model in Supplementary Fig. 10).

## Discussion

A tightly regulated balance between self-renewal and differentiation of cells is critically important for the normal homeostasis of organisms. Cells of the blood lineage are particularly highly reliant on this balance to sustain the hierarchical structure of the hematopoietic system, which gives rise to more than ten distinct mature cell types^39^. Here, we shed light on the molecular cues that regulate self-renewal and differentiation by interrogating the role of chromatin accessibility in safeguarding the identity of blood progenitor cells and their differentiated progeny.

By examining the effects of depleting an essential histone chaperone, CAF-1, we find that restricted access to chromatin plays an important role in (1) preventing spurious differentiation of progenitor cells and (2) ensuring that the identities of differentiated cells are within the scope of the limited potency of the parental progenitor population. We draw these conclusions largely based on our molecular and phenotypic analyses of a genetically stable population of immortalized GMPs (iGMPs), which retain the natural potential to mature into functional granulocytes and macrophages. Specifically, we find that reducing the level of CAF-1 alone arrests self-renewal of iGMPs and sets them on a course of atypical differentiation where cells never mature into specific effector cells. Moreover, the incompletely differentiated CAF-1-depleted cells exhibit hallmarks of multiple lineages (hence their ‘mixed-lineage state’) that are not limited only to the granulocyte and macrophage potency of GMPs but include signatures of the erythroid and megakaryocyte lineages at the progenitor and mature states. Such blurring of the barriers between lineages and stages of differentiation upon depletion of CAF-1 is consistent with increased local chromatin accessibility and facilitated binding of diverse TFs whose activity in normal cells is much more restricted (Supplementary Fig. 10). We speculate that progenitor cells are especially sensitive to aberrant changes in chromatin openness due to a relatively high number of primed genes and expression of transcription factors that stand poised to push the cells down one or another path of differentiation.

Our analyses of chromatin accessibility, transcriptomes, and phenotypes induced by CAF-1 depletion (mixed-lineage state) or HOXA9 inactivation (normal neutrophil differentiation) combined with the characterization of TF binding and activity at specific chromatin sensitive sites identified CEBPA and ELF1 as key regulators of the multi-lineage state. It is worth noting that neither CEBPA or ELF1 stand out in terms of their expression changes during GMP differentiation and would likely have been ignored if judged by expression alone (Supplementary Fig. 7c). Instead, the significance of these TFs during GMP differentiation only became obvious from our analyses of chromatin accessibility changes which highlighted a particular sensitivity of regulatory sites, i.e., promoters and enhancers, identified based on their ‘activating’ histone modifications (H3K4me1, H3K4me3, and H3K27ac; Fig. 5g–i). A large fraction of the HOXA9-sensitive sites already showed an appreciable level of binding by ELF1 and CEBPA in uninduced cells (Fig. 5f, i); upon induction of differentiation, binding of CEBPA and to a lesser extent ELF1 further increased (Fig. 5f, i). A notable exception to this trend was CAF-1 sensitive regulatory sites, which showed essentially no detectable ELF1 binding in uninduced cells, but significant binding upon CAF-1 depletion. Supported by our downstream genetic analyses and the previously documented roles of ELF1 in blood cell differentiation^36,37^, we speculate that this subset of sensitive sites may be critical for the observed induction of the mixed-lineage state by CAF-1 depletion. In contrast, CEBPA binding did not increase at the CAF-1 sensitive sites (Fig. 5f, i) despite its functional importance in the differentiation of CAF-1 OFF cells (Fig. 6e, h, i, l and Supplementary Fig. 9d, e). These results suggest that the loss of CAF-1 indirectly contributes to CEBPA activity.

With regards to the control of chromatin accessibility by CAF-1 and the functional relevance of sensitive sites created by its loss, there are several other observations that would deserve further attention. For instance, a large proportion of CAF-1-sensitive sites appear to be heterochromatic and show no recognition by either ELF1 or CEBPA upon their opening induced by CAF-1 depletion. This, however, does not rule out a potential contribution of these sites to the mixed-lineage phenotype, which might come about through the activation of local coding and non-coding genes by different factors. Furthermore, given the biased chromatin opening and gene activation upon CAF-1 depletion, we focused our analyses on the opened DARs (CAF-1 sensitive sites); however, there is also a significant number of closed DARs, i.e., less accessible sites (Fig. 5a, b). Although the closed DARs likely arise as an indirect consequence, their regulation by CAF-1 could nevertheless have an important impact on TF binding and transcriptional control in GMPs. Thus, future studies are warranted to explore the role of TFs in the differentiation of GMPs and other committed progenitors.

scRNA-seq has deepened our understanding of transcriptional programs that govern lineage commitment and unmasked the heterogeneity even within sorted populations of cells. Consistent with our findings, a study employing scRNA-seq demonstrated that mixed-lineage states do not arise during normal differentiation of myeloid progenitors^5^. Additionally, this same study identified CEBPA as a key regulator in lineage restriction, consistent with our identified role of CEBPA in promoting differentiation of GMPs upon CAF-1 suppression or Hoxa9 inactivation. Based on these results and previous studies of CAF-1-dependent regulation of cellular plasticity^14–16,18–21^, we propose that perturbing CAF-1 may present a broadly applicable approach to discern transcriptional programs that are central to the differentiation of various precursor cells into progressively more lineage-restricted progeny. This is noteworthy since previous studies that investigated the function of CAF-1 in the maintenance of cell identity did not probe the effect of CAF-1 ablation on lineage fidelity in differentiation systems of stem and progenitor cells.

Finally, it remains unclear why depletion of CAF-1 affects certain chromatin regions disproportionately more than others. As a general chromatin assembly factor and regulator of heterochromatin^15^, CAF-1 might be expected to have a substantially more wide-spread and pleiotropic effect. In addition to its canonical function as a histone chaperone, CAF-1 has also been hypothesized to act through the recruitment of chromatin-modifying enzymes that promote gene silencing^15,19,20^. In addition, a recent study proposed that CHAF1B might compete with TF binding without affecting chromatin accessibility^18^. While the CAF-1 interactome in hematopoietic cells has not been characterized, previous studies in continuous cell lines and ESCs have identified a number of CAF-1 interactors most of which are associated with transcriptional silencing, including LSD1, HP1, SETDB1, and HDACs^14^. This contrasts with the binding partners of HOXA9, which have largely been linked to transcriptional activation, for instance, PBX1, PU.1, MLL3/4, or CEBPA^40,41^. These observations along with the canonical functions of CAF-1 and HOXA9 would suggest fundamentally different mechanisms of cell differentiation triggered by the loss of CAF-1 or inactivation of HOXA9, namely, transcriptional de-repression or attenuation of an existing transcriptional program, respectively. In light of our characterization of the histone modifications enriched at CAF-1 sensitive sites, which reveals signatures of enhancer/promoter elements and heterochromatin regions (Fig. 5g–i), it is conceivable that in GMPs the repressive role of CAF-1 could be aided by recruitment of the above silencing molecules, or co-factors, to these sites. It is unclear, however, which of the co-factors might be recruited and to what subset of CAF-1 sensitive sites, in addition to uncertainty about a functional necessity for such recruitment. Therefore, whether CAF-1 achieves its locus specificity exclusively in its capacity as a general histone chaperone and whether its protein subunits might adopt additional roles remain unsolved open questions. Future mechanistic studies of CAF-1 are needed to shed light on the activities of this intriguing complex in development and disease.

## Methods

### In vivo *Chaf1b* excision

All animal studies were performed with approval from the Northwestern University Institutional Animal Care and Use Committee (IACUCU # IS00006115) and conducted in accordance with institutional and national regulatory standards. The *Chaf1b*-targeted C57Bl/6 embryonic stem cells (Chaf1b^tm2a(EUCOMM)Hmgu^) were obtained from EUCOMM (European Conditional Mouse Mutagenesis). These cells were introduced into C57Bl/6 blastocysts to derive chimeras. Two male chimeras were bred to wild-type albino C57Bl/6 females, and pigmented offspring were checked for full insertion of critical portions of the targeting construct by PCR and sanger sequencing. Complete *Chaf1b*-targeted mice were back-crossed to wild-type C57Bl/6 J mice for six additional rounds of breeding. Targeted mice were then bred to C57Bl/6 Flp^+^ mice to create *Chaf1b*^+/fl^ animals. *Mx1*^*+*^*Chaf1b*^*fl/fl*^ mice were generated by crossing to Mx1-Cre mice. To induce Mx1-Cre activity, mice were injected peritoneally with a 200 µL bolus of polyI:C at 12.5 mg/kg in physiological water. C57Bl/6 Flp^+^ (#005703) and Mx1-Cre (#003556) mice strains were obtained from the Jackson Lab. All mice were housed in a controlled environment (20 ± 2 °C, 12/12 h light/dark cycle) in a barrier-grade animal facility. Equal numbers of male and female mice were used. Experimental mice were co-housed with control mice. Mice were euthanized at 8–12 weeks via inhalation of carbon dioxide in a chamber followed by cervical dislocation.

### Cell culture

Immortalized granulocyte-macrophage progenitor-like (iGMP) cells containing *ER-HOXA9*^22,23^ were cultured in RPMI-1640 supplemented with 10% FBS, 100 U mL^−1^ penicillin, 100 µg mL^−1^ streptomycin, 2 mM L-alanyl-L-glutamine dipeptide and stem cell factor (SCF). Depending on the batch, media was made with 1–2% SCF-conditioned media from SCF-secreting Chinese hamster ovary cells (gift from David Sykes Lab). β-Estradiol (E2) was added to a final concentration of 150 ng/mL for normal culture. 293 T packaging cells (CRL-3216, ATCC) were cultured in DMEM supplemented with 10% FBS, 100 U mL^−1^ penicillin, 100 µg mL^−1^ streptomycin, 55 µM β-mercaptoethanol, 2 mM L-alanyl-L-glutamine dipeptide and 1× MEM NEAA. All cells were cultured at 37 °C with 5% CO_2_. All cell lines were routinely tested for mycoplasma contamination. CD117 (17-1171-82, eBioscience) enriched hematopoietic stem and progenitor cells (HSPCs) from mice were cultured in Stemspan Serum Free Expansion Media (SFEM2, 09650, STEMCELL Technologies) supplemented with 50 ng/mL recombinant mouse SCF (78064, STEMCELL Technologies), 10 ng/mL recombinant mouse IL3 (78042, STEMCELL Technologies), 10 ng/mL recombinant mouse IL6 (78052, STEMCELL Technologies), and 1:200 human Low Density Lipoprotein (hLDL; 02698, STEMCELL Technologies).

### Flow cytometry

For live-cell staining, antibodies were diluted 1:200 in FACS buffer (PBS + 5% FBS + 1 mM EDTA) and cells were stained in 100 µL for 20 min at 4 °C, in the dark. After incubation, cells were washed twice with excess FACS buffer and then resuspended in FACS buffer for flow cytometry. Propidium iodide was added before analysis at a final concentration of 1 µg/mL to distinguish dead cells. Mouse antibodies CD11b-PE/Cy7 (#101216), Gr1-PE (#108407), CD41-PE (#133905), CD105-PE (#120407) were purchased from BioLegend, CD11b-PE (12-0112-82) and Gr1-APC (17-5931-82) were purchased from eBioscience. Flow cytometry data were acquired with NovoExpress (v1.5) on NovoCyte 2100Y/Quanteon cytometers at UCR, with FACSDiva (v9.0) on a BD LSR2 cytometer at MGH, and with FACSDiva (v9.0) on a BD Fortessa cytometer at Northwestern University and Cincinnati Children’s Hospital Medical Center. Flow data were analyzed with FlowJo (v9, v10).

### Inducible CAF-1 depletion or HOXA9 inactivation in iGMPs

For LacO-shChaf1b expression (CAF-1 OFF condition), IPTG was added at a concentration of 500 µM to iGMPs cultured with E2 and replenished every two days. For Hoxa9 inactivation (HOXA9 OFF condition), cells were washed with PBS twice to remove E2 and then cultured in complete RPMI media without E2. Cell media was replenished with corresponding media every 2 days during time courses. For the differentiation commitment assay, i.e., “point of no return” experiment, we pulsed cells with IPTG or estradiol withdrawal (−E2) for 24 h increments. After each increment, cells were left to recover in self-renewing media condition, without induction (−IPTG, + E2), until 144 h post-initial treatment. See respective (pulse+chase) conditions in the experimental outline in Fig. 2e. All timepoints, except (0 + 6 days), were induced with IPTG or −E2 on Day 0 with corresponding pulse periods (24, 48, 72, 96, and 120 h) and then returned to self-renewing media conditions. After 144 h (Day 6), all timepoints were collected and analyzed by flow cytometry for Lys-GFP. (see Flow Cytometry in Methods). A similar strategy of IPTG/E2 treatment was used for the sequential pulses and combined conditions (see experimental outlines in Fig. 3h and Supplementary Fig. 5a).

### Growth curve

Growth curves were performed using ViaLight Plus Cell Proliferation and Cytotoxicity BioAssay Kit (LT07-121, Lonza) according to the supplier’s instruction. Cells were seeded at 5000 cells per well in a 96-well plate. At each timepoint, cells were lysed for 10 min and then incubated with ATP monitoring reagent plus for 2 min. Luminescence was measured with a Promega GloMax-Multi Detection System with internal software (v1.1.14, # E8916).

### Cell cycle analysis

Cell cycle analysis was performed using the Click-iT Plus EdU kit (Thermo-C10646) following the manufacturer’s recommendations. iGMPs prior to and post-induction of HOXA9 OFF or CAF-1 OFF for 48 h were pulse-labelled with 10 uM EdU for 1 h. One million cells were collected and fixed with 4% formaldehyde and permeabilized with a saponin-based wash. EdU was labeled in a reaction cocktail with Alexa Fluor 594 picolyl azide for 30 min at room temperature. After labeling, total DNA content was stained with 250 ng/mL DAPI overnight at 4 °C. Cells were then analyzed by flow cytometry in PBS with 5% FBS + 1 mM EDTA.

### Growth arrest

For uninduced cells, growth arrest was achieved by depletion of SCF at 0.1× and 0.02× concentrations and compared to self-renewal culture conditions (1× SCF; see Supplementary Fig. 3f). For the CAF-1 OFF condition, cells were pretreated with 500 µM IPTG for 12 h and then transferred into media containing either 1×, 0.1×, or 0.02× SCF for an additional 60 h and analyzed by flow cytometry 72 h post-induction (Supplementary Fig. 3f). For the HOXA9 OFF condition, cells were transferred into either 1×, 0.1×, or 0.02× SCF-containing media for 12 h followed by E2 withdrawal and subsequently cultured in their respective SCF concentrations for an additional 48 h and analyzed by flow cytometry 60 h post-growth arrest (Supplementary Fig. 3f). All conditions were seeded at the same cell concentration at the beginning of each treatment (6.0 × 10^4^ cells/mL).

### RT-qPCR

RNA was extracted with the Direct-zol RNA miniprep plus kit (Zymo Research- R2072) and then reverse-transcribed with PrimeScript RT Master Mix (Takara-RR036A) or the SuperScript VILO cDNA Synthesis Kit (ThermoFisher-11754050) according to the supplier’s instruction. Quantitative PCR was performed using the PowerUp SYBR Green master mix (ThermoFisher-A25741) in a BIO-RAD CFX connect cycler with CFX Maestro (v2.2) software. Primers are listed in Supplementary Table 1. Fold change mRNA results are presented as 2^-ΔΔCt^ values normalized to the expression of β-actin or Gapdh and relative to negative control samples. All reactions were performed in technical triplicates. The means and standard deviations were calculated in GraphPad Prism (v8.0, v9.2) software.

### SDS-PAGE and western blot analysis

Whole-cell lysates were run on 10 or 15% SDS-polyacrylamide gels and transferred to nitrocellulose membrane (1620097, Bio-Rad) for 1–2 h at 145 V, 4 °C. Membranes were blocked for 1 h in 5% non-fat dry milk in 1 ×TBS with 0.1% Tween-20 (TBST), washed three times in TBST for 5 min., and incubated overnight at 4 °C with primary antibody diluted 1:500–1:1000 in 3% BSA in TBST. The following primary antibodies were used: anti-CHAF1A (sc-10206, Santa Cruz, discontinued), anti-CHAF1B (sc-393662, Santa Cruz), anti-TBP (ab818, Abcam), anti-CEBPα (8178, Cell Signaling), anti-ELF1(sc-133096, Santa Cruz), anti-HOXA9 (NBP2-32356, Novus Biological), anti-ER (8644T, Cell Signaling), anti-TUBULIN (2144S, Cell Signaling). Blots were washed in TBST, incubated with HRP-conjugated secondary antibodies at 1:2000 dilution (anti-Mouse, Millipore Sigma-AP124P; anti-Rabbit, Millipore Sigma-AP307P) in 5% non-fat milk in TBST for 1 h at room temperature and blots were washed three times in TBST for 5 min. HRP signal was detected by Western Lightning Plus-ECL (NEL103E001EA, Perkin Elmer). Actin was detected with HRP-conjugated primary antibody (A3854, Millipore Sigma) diluted 1:20,000 in TBST with 5% non-fat milk and incubated for 15–30 min at room temperature. ImageJ (v1.53c) was used for relative densitometric quantifications displayed below blots. Relative values are normalized to ACTIN, TUBULIN or TBP. ACTIN, TUBULIN, and TBP are presented as, at minimum, sample processing controls in the figures. Uncropped versions of all presented blots are included with respective loading controls in the Source Data file.

### Cytospins and Wright-Giemsa staining

Cells were prepared in PBS at a concentration of ~2 million/mL. Cytospin (Thermo Scientific Shandon) preparations were made (1000 rpm, 60 s), and the cells were allowed to air dry. Cells were stained in 100% Wright-Giemsa (Siemens) for 2 min, and in 20% Wright-Giemsa diluted in the buffer for 12 min. Stained cells were rinsed in deionized water, and coverslips were affixed with Permount prior to microscopy. Images were acquired using Nikon Elements (v5.11.01).

### Phagocytosis assay

HOXA9 iGMPs were differentiated out of E2 for a period of 96 h. Cells were resuspended in media along with fluorescein-labeled heat-killed *Escherichia coli* BioParticles (pHrodo, Molecular Probes). Cells and bioparticles were agitated at 37 °C for 60 min prior to flow cytometry; DAPI was used as a viability dye.

### Plasmids and lentivirus production

For inducible CAF-1 depletion, Chaf1b-targeting shRNAs were cloned into the pLKO-TRC912 1X LacI IPTG-inducible vector (Broad institute) harboring a puromycin-selectable marker. For constitutive CAF-1 depletion Chaf1b-targeting shRNAs were cloned into pLKO-TRC025 vector (Broad institute) harboring a puromycin-selectable marker. For the TF loss of function experiments, shRNA targeting C/EBPα and ELF1 were cloned into pLKO.1 vector (Addgene, plasmid # 26655) harboring a blasticidin-selectable marker. See Supplementary Table 1 for shRNA sequences. For IPTG induction, a control vector harboring a stuffer sequence was used. For the constitutive knockdown of Chaf1b or TFs, control vectors harboring a luciferase targeting shRNA or scrambled control shRNA were used, respectively. See Supplementary Table 1 for shRNA sequences. Single guide RNAs (sgRNAs) targeting the CEBPA and ELF1 locus were cloned into lentiCRISPR v2 vector (Addgene, plasmid #83480) harboring the wild-type Cas9 coding region, an sgRNA expression cassette, and a blasticidin-selectable marker. See Supplementary Table 1 for guide sequences. RNAi-resistant Chaf1b cDNA was cloned in the retroviral MSCV backbone (Clontech). Retroviruses from MSCV and MIGR1 vectors (Addgene, plasmid #27490) were produced as described previously^14,18^. Lentiviruses were produced by transfection of 293 T cells with Δ8.9 and VSVG plasmids. The virus was harvested at 36, 60, and 84 h post-transfection and precipitated using PEG3500 (Sigma-Aldrich, cat#P4338). For transduction, 150,000 cells were plated per well in a 12-well dish, and spin-infected at 2500 rpm for 1.5 h in the presence of 10 µg mL^−1^ of polybrene (Millipore). After 48 h, transduced cells were selected with either 5 µg mL^−1^ puromycin or 10 µg mL^−1^ blasticidin (Gibco) for 6 days.

### 10x chromium single-cell RNA sequencing

iGMP cells were collected at 0, 48, and 96 h upon CAF-1 suppression or HOXA9 inactivation. Live cells were sorted for each condition and checked for differentiation status and viability post sorting following the 10X genomics guidelines. scRNA-seq libraries were then prepared following the instruction manual of 10X Chromium (v2). The cDNA library and final library after index preparation were checked on a bioanalyzer (High Sensitivity DNA reagents, Agilent Technology) for quality control. Following the library preparation, the sequencing was performed with paired-end sequencing of 75nt each end on HiSeq4000, by Novogene, Inc. Cells were sequenced to an average depth of 100,000 reads per cell.

### 10x chromium single-cell RNA-seq bioinformatics analysis

The raw reads were mapped to GRCm38 using a standard CellRanger (v3.1.0) pipeline. The R package Seurat (v3.0.1) was used for further analysis. Cells with less than 200 genes or more than 6000 genes (potential doublets) were removed, and only genes that were expressed in at least three cells were kept in the analysis. Cells with mitochondrial gene percentage >10% were filtered out. Unsupervised clustering analysis of 10X scRNA-seq data was performed after normalizing for total reads per cell and log-transformed.

### Bulk RNA-seq

Total RNA from cells was DNase treated and purified using Qiagen RNeasy mini kit (according to manufacturer’s instructions). RNA quality was assessed using an Agilent 2100 Bioanalyzer. RNA-seq libraries were prepared with NEBNext UltraDirectional kit (New England Biolabs) for iGMPs and TruSeq Stranded Total RNA kit (Illumina) for HSPCs. Libraries were pooled and sequenced on Illumina HiSeq 2500 instrument. On average 28 million reads were generated per library.

### Bulk RNA-seq data analysis

STAR aligner (v2.7)^42^ was used to map sequencing reads to transcripts in the mouse mm9 reference genome. Read counts for individual transcripts were produced with HTSeq-count (v0.11.1)^43^, followed by the estimation of expression values and detection of DEGs using EdgeR(v3.14)^44^. DEGs were defined by at least 1.5-fold change with FDR less than 0.05. A more stringent 2-fold change and FDR less than 0.01 cutoffs were applied for the correlation analysis with ATAC-seq peaks (see below and legends). GSEA (v4.2)^31^ was used to analyze the enrichment of functional gene groups among differentially expressed genes. Signal tracks are presented with the IGV genome browser (v2.9.4).

### Bulk ATAC-seq

To generate ATAC-seq libraries, 50,000 cells were used and libraries were constructed as previously described^45^. Briefly, cells were washed in PBS twice, counted and nuclei were isolated from 100,000 cells using 100 μl hypotonic buffer (10 mM Tris-HCl pH 7.4, 10 mM NaCl, 3 mM MgCl_2_, 0.1% NP40) to generate two independent transposition reactions. Nuclei were split in half and treated with 2.5 μl Tn5 Transposase (Illumina) for 30 min at 37 °C. DNA from transposed nuclei was then isolated and PCR-amplified using barcoded Nextera primers (Illumina). Library quality control and quantitation were carried out using high-sensitivity DNA Bioanalyzer and Qubit, followed by paired-end sequencing (PE50) on the Illumina HiSeq 2500 instrument.

### Bulk ATAC-seq data analysis

Sequencing reads were aligned against the mm9 reference genome using BWA (v0.7.17)^46^. Reads mapping to the mitochondrial genome and duplicate reads were removed. Peaks were called using the HOTSPOT method^47^. Read numbers and densities were calculated across all samples for each genomic region among the union of all peaks in all samples. Regions of differential accessibility (DARs) were identified using edgeR (v3.14)^44^, with the cutoffs of at least 2-fold difference and FDR < 0.01. Sequence motifs enriched among these differentially accessible regions were identified using MEME-ChIP (v5.4.1)^32^. To determine the fractions of opened or closed DARs located within 100 kb of up- or downregulated genes in each condition (CAF-1 OFF and HOXA9 OFF), bedtools (v2.29.2) window was used with -u -w 100,000 parameters and gene body regions. Additionally, for each set of DARs (Opened and Closed DARs in CAF-1 OFF or HOXA9 OFF conditions) the fractions of random regions overlapping an up- or downregulated gene in each condition were calculated using 100 sets of shuffled regions of the same size and number. The average and standard deviation of these fractions were used to calculate the Z-scores and *p*-values. To define candidate enhancers and promoters within the sensitive sites, open DARs were first filtered based on fold enrichment of H3K4me1 and H3K4me3 over a 4 kb region centered on each site using deepTools (v3.5.0) computeMatrix. A threshold of more than 3-fold enrichment for H3K4me1 and 0.05-fold enrichment for H3K4me3 was chosen. Subsequently, these regions were classified as promoters if located within 2 kb upstream or 500 bp downstream of annotated transcription start sites. All remaining regions with enriched H3K4me1 or H3K4me3 were classified as enhancers. Signal tracks are presented with the IGV genome browser (v2.9.4).

### ChIP-seq

To construct ChIP-seq libraries 10 million cells were collected from respective conditions (either uninduced or 48 h post CAF-1 OFF and HOXA9 OFF inductions). Cells were then fixed in culture media with 4% formaldehyde for 15 minutes at room temperature. After fixing cells were washed with DPBS and the cell pellet was flash-frozen in liquid nitrogen and stored at −80 °C.

Nuclei were isolated by lysing cells with cell lysis buffer (20 mM Tris-HCl pH 8.0, 85 mM KCl, 0.5% NP40) for 10 minutes at 4 °C and spinning at 12,000 RPM for 4 min at 4 °C. Nuclei were then lysed for 10 min at 4 °C in nuclear lysis buffer (10 mM Tris-HCl pH 7.5, 1% NP40, 0.5% Na Deoxycholate, 0.1% SDS). Lysed nuclei were then sonicated using a Branson 250 probe sonifier at 40% amplitude for 5 minutes in an ice bath to garner 50% of fragments below 1000 bp, and less than 10% under 200 bp. Chromatin was stored at −80 °C while confirming input fragment distribution by gel electrophoresis. After confirmation of fragment distribution, immunoprecipitations were carried out in nuclear lysis buffer supplemented with 167 mM NaCl and 1.2 mM EDTA. Protein A or G beads (for rabbit or mouse antibodies, respectively; 10002D/10004D, ThermoFisher) preconjugated with 10 µg (TFs) or 2 µg (histone marks) of antibody were added to the sheared chromatin for 2 h at 4 °C for each target. The following antibodies were used CEBPA (8178BF, Cell Signaling), ELF1 (A301-443A, Bethyl), H3K4me3 (ab8580, Abcam), and H3K27ac (ab4729, Abcam). Immunoprecipitations were washed sequentially two times each with low salt buffer (0.1% DOC, 0.1% SDS, 1% Triton X-100, 140 mM NaCl, 1 mM EDTA, 20 mM Tris-HCl pH 8.0), high salt buffer (0.1% DOC, 0.1%SDS, 1% Triton X-100, 500 mM NaCl, 1 mM EDTA, 20 mM Tris-HCl pH 8.0), LiCl buffer (0.25 M LiCl, 1% NP40, 1% Na Deoxycholate, 1 mM EDTA, 10 mM Tris-HCl pH 8.0), and TE (10 mM Tris-HCl pH 8.0, 1 mM EDTA pH 8.0) and then eluted in elution buffer (10 mM Tris-HCl pH 8.0, 5 mM EDTA, 300 mM NaCl, 0.1% SDS). Immunoprecipitations were reverse crosslinked overnight at 65 °C after adding 7.25× reverse crosslinking buffer (250 mM Tris-HCl pH 6.5, 1.25 M NaCl, 62.5 mM EDTA, 5 mg/ml Proteinase K [EO0491, ThermoFisher], 62.5 ug/ml RNase A [EN0531, ThermoFisher]). Input samples were reverse crosslinked by diluting sheared chromatin 1:5 with water and adding 7.25× reverse crosslinking buffer overnight at 65 °C. Reverse-crosslinked DNA was isolated with 1.8x AMPure XP (A63880, Beckman Coulter) beads and used for library preparation.

NEB Ultra II DNA Library Prep and Index Kits (E7645, E6440) were used according to the manufacturer’s protocol with modifications. Briefly, up to 10 ng DNA was used for end preparation and adapter ligation without size selection. NEB indexes were added with 2-step PCR using an optimized number of cycles. Fragment distribution of prepared libraries was confirmed by Bioanalyzer to contain over 90% fragments in the 200–600 bp range and then submitted to Illumina Novaseq paired-end sequencing at the University of California Irvine Genomics High-Throughput Facility.

### ChIP-seq data analysis

Base-calling and demultiplexing was done by the sequencing center and sequence quality was confirmed (NVCS/RTA v1.7/v3.4.4). Reads were mapped to mm9 (bowtie2, v2.2.9), duplicates were removed (bamUtil, v1.0.14) and peaks were called with MACS2 (v2.2.6) using all default parameters for paired-end sequencing. CPM normalized bigwig files were generated with deepTools (v3.5.0) and used to generate heatmaps and meta-analysis profiles. Signal tracks are presented with the IGV genome browser (v2.9.4). Fold change peak enrichment (H3K27ac, H3K4me3) was determined by using bedmap (v2.4.38) to calculate the number of called ChIP-seq peaks overlapped (by at least 1 bp) with CAF-1 and HOXA9-sensitive sites or with shuffled peaks of the same size and quantity (bedtools v2.29.2).

### CUT&RUN

CUT&RUN assays were performed as previously described in uninduced iGMPs^48–50^. In summary, 0.5 million cells were collected for each sample. Cells were washed twice (20 mM HEPES pH 7.5, 150 mM NaCl, 0.5 mM spermidine, protease inhibitor) and bound to concavalin A beads (EpiCypher 21-1401).  Cells were then permeabilized and incubated overnight at 4 °C with primary antibody at a 1:100 dilution (wash buffer + 0.01% digitonin, 2 mM EDTA). The following antibodies were used H3K4me1 (ab8895, Abcam), H3K27me3 (9733S, Cell Signaling), H3K9me3 (ab8898, Abcam), and IgG (2792S, Cell Signaling). Cells were then washed twice with 0.01% digitonin in wash buffer. pAG-MNase (15–1116, EpiCypher) was then used at 1:20 dilution for each 50 μL CUT&RUN reaction and incubated for 10 min at room temperature. Cells were then washed twice with digitonin buffer and 2 mM CaCl_2_ was added for 2 h at 4 °C. Stop buffer (340 mM NaCl, 20 mM EDTA, 4 mM EGTA, 50 µg/mL RNase A, 50 µg/mL glycogen) was added at 1.5× dilution and incubated for 10 min at 37 °C. DNA was purified with CUTANA DNA purification kit (EpiCypher, 14-0050). Library construction was performed using the NEBNext UltraII DNA Library Prep Kit from NEB (E7645S). Indexed samples were run using the Illumina HiSeq 4000 platform with paired-end sequencing.

### CUT&RUN data analysis

CUT&RUN data analysis was performed as previously described with modifications^51^. Briefly, raw reads were subjected to adapter removal (cutadapt v2.10) and mapped to genome mm9 (bowtie2 v2.4.1) using --end-to-end --very-sensitive -–no-mixed -–no-discordant --phred33 -I 10 -X 700 parameters. Duplicated reads were removed using Picard MarkDuplicates tools (v2.23.3). Bigwig files were generated by deepTools (v3.3.0) bamCoverage and used for visualization in IGV (v2.9.4). CUT&RUN peaks were called using SEACR software (v1.3) in relaxed mode with normalization to IgG control^52^. Fold change peak enrichment (H3K4me1, H3K27me3, and H3K9me3) was determined by using bedmap (v2.4.38) to calculate the number of called CUT&RUN peaks overlapped (by at least 1 bp) with CAF-1 and HOXA9-sensitive sites or with shuffled peaks of the same size and quantity (bedtools v2.29.2).

### Statistical analysis

Statistical tests are reported in the figure legends. p-values are given within the figures where significance is reported. *p*-values >0.0001 are given exactly and (*) denotes *p*-values <0.0001. Prism (Graphpad, v9.2.0) and R (v3.6) were used for statistical analyses. Appropriate statistical tests were determined using Prism’s recommendations based on the number of parameters and experimental groups tested.

### Reporting summary

Further information on research design is available in the Nature Research Reporting Summary linked to this article.

## Supplementary information

Supplementary Information

Description of Additional Supplementary Files

Supplementary Data 1

Reporting Summary

## Data Availability

The scRNA-seq, RNA-seq, ATAC-seq, CUT&RUN, and ChIP-seq data generated in this study have been deposited in the Gene Expression Omnibus (GEO) database and are publicly available under the super series accession number GSE158229. Genome assemblies used in this paper (GRCm38/mm10, GCF_000001635.2, and MGSCv37/mm9, GCF_000001635.18) are publicly available. Uncropped immunoblots and source data underlying all graphs are presented in the “Source Data” file. Additional RNA-seq data analysis supporting the findings of this study are presented in a Supplementary Data file. The reporting summary for this article is available under supplementary information. Source data are provided with this paper.

## Source data

Source Data

## References

[1] Laurenti E, Gottgens B (2018). From haematopoietic stem cells to complex differentiation landscapes. Nature.

[2] Carrelha J (2018). Hierarchically related lineage-restricted fates of multipotent haematopoietic stem cells. Nature.

[3] Jacobsen SEW, Nerlov C (2019). Haematopoiesis in the era of advanced single-cell technologies. Nat. Cell Biol..

[4] Liggett LA, Sankaran VG (2020). Unraveling hematopoiesis through the lens of genomics. Cell.

[5] Paul F (2016). Transcriptional heterogeneity and lineage commitment in myeloid progenitors. Cell.

[6] Zhang Y, Gao S, Xia J, Liu F (2018). Hematopoietic hierarchy—an updated roadmap. Trends Cell Biol..

[7] Loughran SJ, Haas S, Wilkinson AC, Klein AM, Brand M (2020). Lineage commitment of hematopoietic stem cells and progenitors: insights from recent single cell and lineage tracing technologies. Exp. Hematol..

[8] Heyworth C, Pearson S, May G, Enver T (2002). Transcription factor-mediated lineage switching reveals plasticity in primary committed progenitor cells. EMBO J..

[9] Xie H, Ye M, Feng R, Graf T (2004). Stepwise reprogramming of B cells into macrophages. Cell.

[10] Atlasi Y, Stunnenberg HG (2017). The interplay of epigenetic marks during stem cell differentiation and development. Nat. Rev. Genet..

[11] Hammond CM, Stromme CB, Huang H, Patel DJ, Groth A (2017). Histone chaperone networks shaping chromatin function. Nat. Rev. Mol. Cell Biol..

[12] Franklin R, Murn J, Cheloufi S (2021). Cell fate decisions in the wake of histone H3 deposition. Front. Cell Dev. Biol..

[13] Kolundzic E (2018). FACT sets a barrier for cell fate reprogramming in caenorhabditis elegans and human cells. Dev. Cell.

[14] Cheloufi S (2015). The histone chaperone CAF-1 safeguards somatic cell identity. Nature.

[15] Cheloufi S, Hochedlinger K (2017). Emerging roles of the histone chaperone CAF-1 in cellular plasticity. Curr. Opin. Genet. Dev..

[16] Ishiuchi T (2015). Early embryonic-like cells are induced by downregulating replication-dependent chromatin assembly. Nat. Struct. Mol. Biol..

[17] Houlard M (2006). CAF-1 is essential for heterochromatin organization in pluripotent embryonic cells. PLoS Genet..

[18] Volk A (2018). A CHAF1B-Dependent Molecular Switch in Hematopoiesis and Leukemia Pathogenesis. Cancer Cell.

[19] Cheng L (2019). Chromatin Assembly Factor 1 (CAF-1) facilitates the establishment of facultative heterochromatin during pluripotency exit. Nucleic Acids Res..

[20] Ng C (2019). The histone chaperone CAF-1 cooperates with the DNA methyltransferases to maintain Cd4 silencing in cytotoxic T cells. Genes Dev..

[21] Yang BX (2015). Systematic identification of factors for provirus silencing in embryonic stem cells. Cell.

[22] Sykes DB (2016). Inhibition of dihydroorotate dehydrogenase overcomes differentiation blockade in acute myeloid leukemia. Cell.

[23] Wang GG (2006). Quantitative production of macrophages or neutrophils ex vivo using conditional Hoxb8. Nat. Methods.

[24] Faust N, Varas F, Kelly LM, Heck S, Graf T (2000). Insertion of enhanced green fluorescent protein into the lysozyme gene creates mice with green fluorescent granulocytes and macrophages. Blood.

[25] Xie X (2020). Single-cell transcriptome profiling reveals neutrophil heterogeneity in homeostasis and infection. Nat. Immunol..

[26] Ruijtenberg S, van den Heuvel S (2016). Coordinating cell proliferation and differentiation: antagonism between cell cycle regulators and cell type-specific gene expression. Cell Cycle.

[27] Smith S, Stillman B (1989). Purification and characterization of CAF-I, a human cell factor required for chromatin assembly during DNA replication in vitro. Cell.

[28] Quivy JP, Gerard A, Cook AJ, Roche D, Almouzni G (2008). The HP1-p150/CAF-1 interaction is required for pericentric heterochromatin replication and S-phase progression in mouse cells. Nat. Struct. Mol. Biol..

[29] Blagosklonny, M. V. & Pardee, A. B. The Restriction Point of the Cell Cycle. Madame Curie Bioscience Database [Internet] (Landes Bioscience, 2013).

[30] Becht, E. et al. Dimensionality reduction for visualizing single-cell data using UMAP. *Nat. Biotechnol.*10.1038/nbt.4314 (2018). 10.1038/nbt.4314 30531897

[31] Subramanian A (2005). Gene set enrichment analysis: a knowledge-based approach for interpreting genome-wide expression profiles. Proc. Natl Acad. Sci. USA.

[32] Machanick P, Bailey TL (2011). MEME-ChIP: motif analysis of large DNA datasets. Bioinformatics.

[33] Pundhir S (2018). Enhancer and transcription factor dynamics during myeloid differentiation reveal an early differentiation block in cebpa null progenitors. Cell Rep..

[34] Rosenbauer F, Tenen DG (2007). Transcription factors in myeloid development: balancing differentiation with transformation. Nat. Rev. Immunol..

[35] Zhang DE (1997). Absence of granulocyte colony-stimulating factor signaling and neutrophil development in CCAAT enhancer binding protein alpha-deficient mice. Proc. Natl Acad. Sci. USA.

[36] Gallant S, Gilkeson G (2006). ETS transcription factors and regulation of immunity. Arch. Immunol. Ther. Exp. (Warsz.).

[37] Suico MA, Shuto T, Kai H (2017). Roles and regulations of the ETS transcription factor ELF4/MEF. J. Mol. Cell Biol..

[38] Bagger FO, Kinalis S, Rapin N (2019). BloodSpot: a database of healthy and malignant haematopoiesis updated with purified and single cell mRNA sequencing profiles. Nucleic Acids Res..

[39] Seita J, Weissman IL (2010). Hematopoietic stem cell: self-renewal versus differentiation. Wiley Interdiscip. Rev. Syst. Biol. Med.

[40] Schnabel CA, Jacobs Y, Cleary ML (2000). HoxA9-mediated immortalization of myeloid progenitors requires functional interactions with TALE cofactors Pbx and Meis. Oncogene.

[41] Sun Y (2018). HOXA9 reprograms the enhancer landscape to promote leukemogenesis. Cancer Cell.

[42] Dobin A (2013). STAR: ultrafast universal RNA-seq aligner. Bioinformatics.

[43] Anders S, Pyl PT, Huber W (2015). HTSeq-a Python framework to work with high-throughput sequencing data. Bioinformatics.

[44] Robinson MD, McCarthy DJ, Smyth GK (2010). edgeR: a Bioconductor package for differential expression analysis of digital gene expression data. Bioinformatics.

[45] Buenrostro JD, Giresi PG, Zaba LC, Chang HY, Greenleaf WJ (2013). Transposition of native chromatin for fast and sensitive epigenomic profiling of open chromatin, DNA-binding proteins and nucleosome position. Nat. Methods.

[46] Li H, Durbin R (2010). Fast and accurate long-read alignment with Burrows-Wheeler transform. Bioinformatics.

[47] John S (2011). Chromatin accessibility pre-determines glucocorticoid receptor binding patterns. Nat. Genet..

[48] Skene PJ, Henikoff JG, Henikoff S (2018). Targeted in situ genome-wide profiling with high efficiency for low cell numbers. Nat. Protoc..

[49] Skene, P. J. & Henikoff, S. An efficient targeted nuclease strategy for high-resolution mapping of DNA binding sites. *Elife*10.7554/eLife.21856 (2017). 10.7554/eLife.21856 PMC5310842 28079019

[50] Zhang, H. et al. Functional interrogation of HOXA9 regulome in MLLr leukemia via reporter-based CRISPR/Cas9 screen. *Elife*10.7554/eLife.57858 (2020). 10.7554/eLife.57858 PMC7599066 33001025

[51] Tang Y (2021). Inhibition of EZH2 primes the cardiac gene activation via removal of epigenetic repression during human direct cardiac reprogramming. Stem Cell Res.

[52] Meers MP, Tenenbaum D, Henikoff S (2019). Peak calling by Sparse Enrichment Analysis for CUT&RUN chromatin profiling. Epigenetics Chromatin.

