# Supplementary material for: Regulation of chromatin accessibility by the histone chaperone CAF-1 sustains lineage fidelity
Source: Nat Commun. 2022 Apr 29;13:2350. doi: 10.1038/s41467-022-29730-6 (PMC9054786; doi:10.1038/s41467-022-29730-6)
Supplement: Supplementary file 1 — Supplementary Information [file 41467_2022_29730_MOESM1_ESM.pdf]

Regulation of Chromatin Accessibility by the Histone Chaperone CAF-1 Sustains Lineage Fidelity

Reuben Franklin, Yiming Guo, Shiyang He, Meijuan Chen, Fei Ji, Xinyue Zhou, David Frankhouser, Brian T. Do, Carmen Chiem, MiHyun Jang, M. Andres Blanco, Matthew G Vander Heiden, Russell C. Rockne, Maria Ninova, David B. Sykes, Konrad Hochedlinger, Rui Lu, Ruslan I. Sadreyev, Jernej Murn, Andrew Volk, Sihem Cheloufi

Supplementary Information

Supplementary information includes 10 Supplementary Figures and 1 Supplementary Table.

Supplementary Figure 1

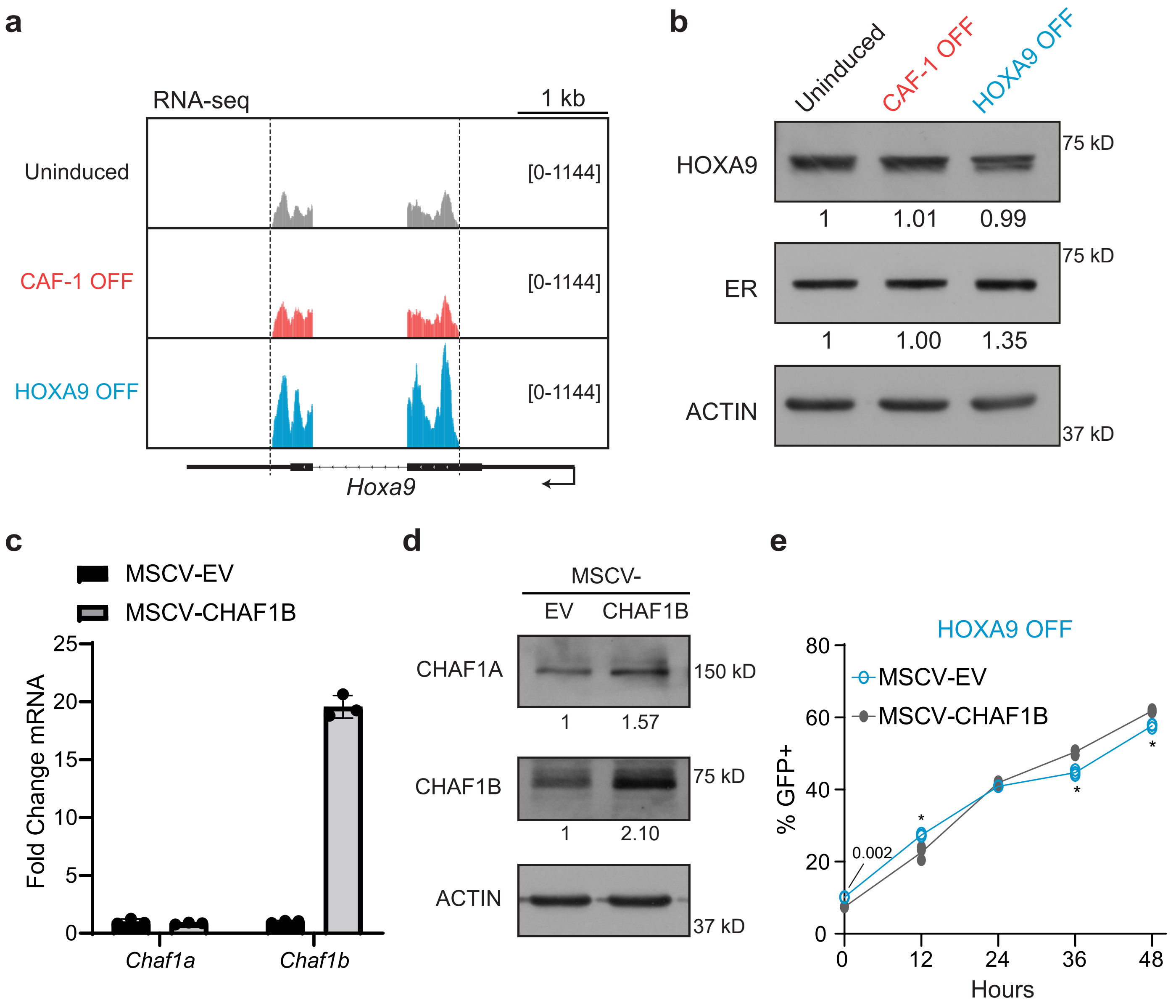

Supplementary Figure 1. HOXA9 expression analysis and CHAF1B overexpression (Related to Figure 1)

a, Representative genome browser snapshot for RNA-seq tracks over the *Hoxa9* locus in uninduced iGMPs and 48 h post CAF-1 OFF and HOXA9 OFF inductions. All conditions show lack of read coverage at the 5'UTR and 3'UTR (dotted lines) of *Hoxa9* indicating expression of the ER-Hoxa9 transgene but not the endogenous *Hoxa9*. RNA-seq tracks from two independent sub-clones were examined showing similar results. **b,** Western blot analysis of HOXA9 in uninduced iGMPs and 48 h post CAF-1 OFF and HOXA9 OFF induction. Membranes were probed with HOXA9 and ER antibodies. Two independent experiments were performed with similar results but only one representative is shown. **c-d,** RT-qPCR and western blot analysis in inducible Chaf1b shRNA HOXA9 iGMPs transduced with either MSCV empty control vector (MSCV-EV) or MSCV vector overexpressing shRNA-resistant CHAF1B (MSCV-CHAF1B) lentiviral vectors. The mean and standard deviation of mRNA fold change expression for three technical replicates is shown in **(c)**. Two independent experiments were performed with similar results, but one representative experiment is shown in (c-d). **e**, Time course flow cytometric analysis of iGMPs analyzed in **(c-d)** for every 12 h within 48 h of HOXA9 inactivation. The mean and standard deviation of percent GFP+ population is plotted from n=3 experimental replicates comparing iGMPs overexpressing CHAF1B with empty vector controls. Two-way ANOVA with Sidak's correction. * $p<0.0001$. Source data are provided as a Source Data file.

Supplementary Figure 2

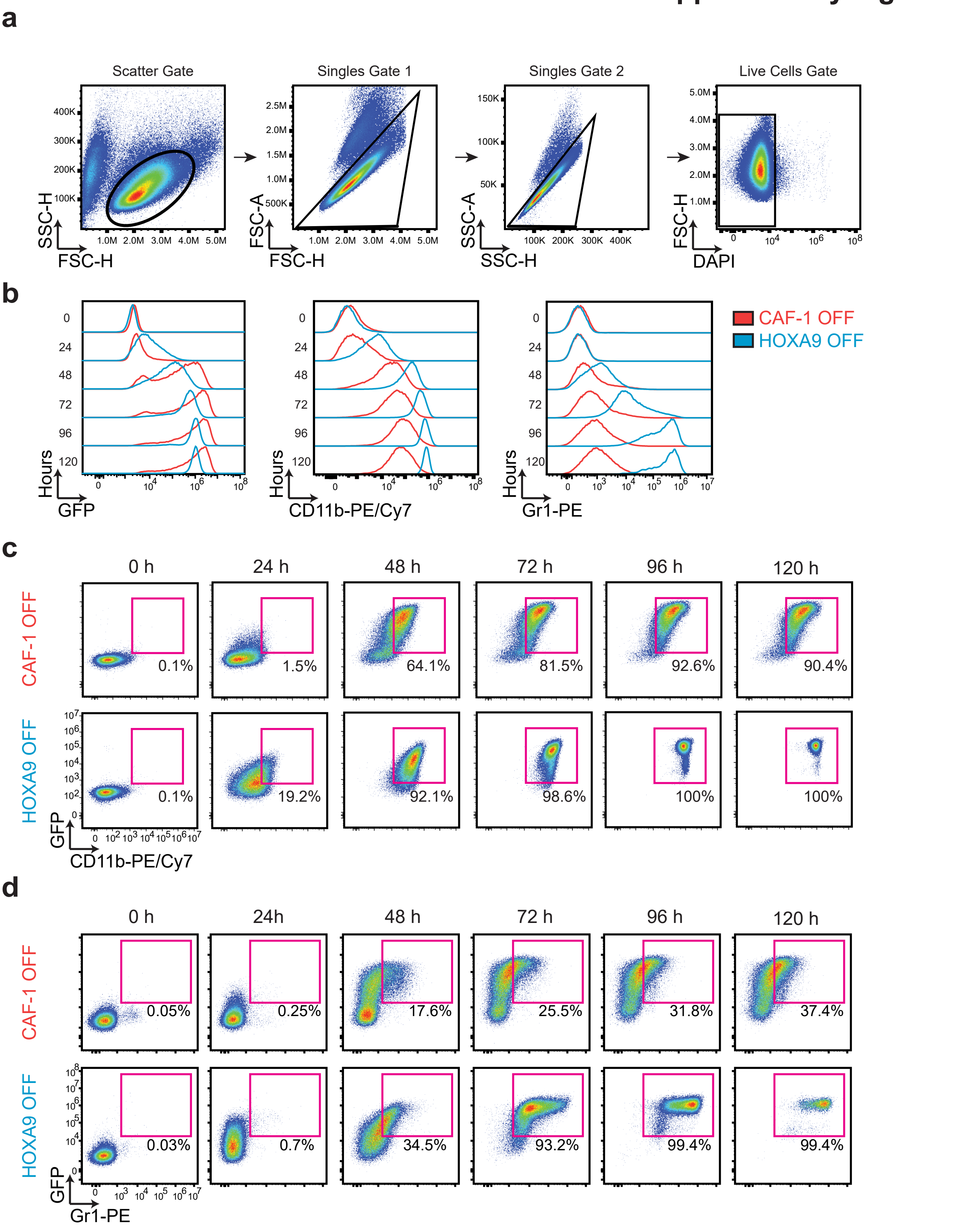

Supplementary Figure 2. Myeloid differentiation kinetics in CAF-1 OFF and HOXA9 OFF iGMPs (Related to Figure 2)

a-d, Time course flow cytometric analysis of myeloid differentiation markers in CAF-1 OFF and HOXA9 OFF iGMPs. **a,** Representative gating strategy of live single cells. **b-d,** Representative histograms and density plots for GFP, CD11b, and Gr1 activation upon differentiation in both conditions. GFP/CD11b and GFP/Gr1 double positive populations are gated in **(c-d)**. See corresponding quantification for each marker in Fig. 2c. Three independent experiments were performed with similar results but only one representative is shown.

Supplementary Figure 3

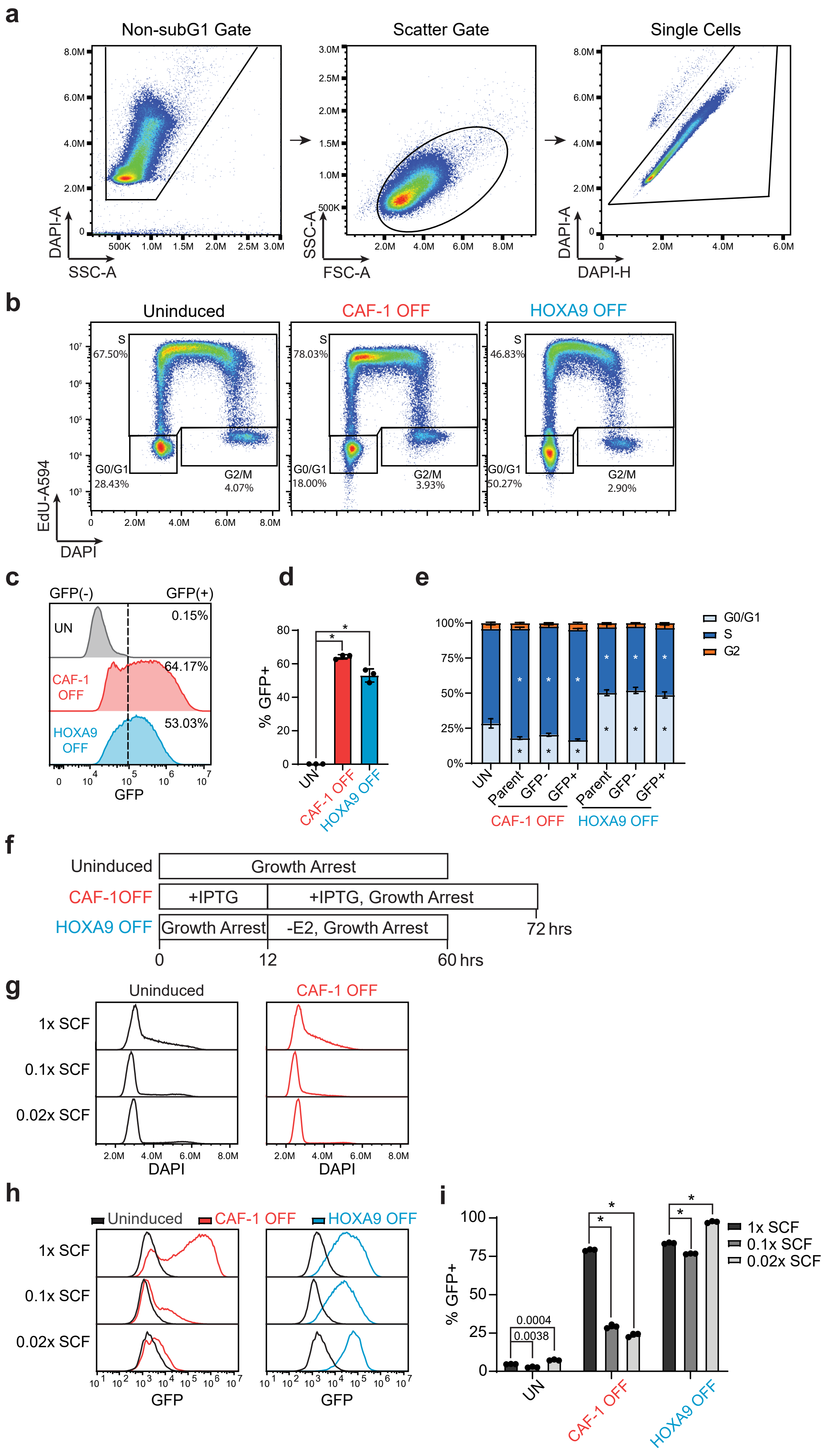

Supplementary Figure 3. CAF-1 depletion mediated differentiation depends on cell cycle progression (Related to Figure 2)

a-e, Cell cycle analysis of uninduced iGMPs (UN) and CAF-1 OFF or HOXA9 OFF iGMPs 48 h post induction. **a-b**, Representative flow cytometric gating strategy **(a)** and analysis **(b)** of cell cycle profiles showing EdU incorporation versus DNA content. G1/G0, S, and G2/M phases of the cell cycle are gated. A594, Alexa Fluor 594 **c,** Histogram of GFP signal for cells analyzed in **(b)** with GFP positive cell gating. **d**, Quantification of data shown in **(c)**. **d-e,** Mean and standard deviation of percent gated populations across all conditions from n=3 experimental replicates. One-way ANOVA with Dunnett's test for **(d)** and Two-way ANOVA with Tukey's test for **(e)**. **e,** Cell cycle phases quantification is shown for ungated parental, GFP negative, and GFP positive populations (shown in **c**) in CAF-1 OFF and HOXA9 OFF conditions. **f-i,** The effect of induced growth arrest by SCF withdrawal in uninduced, CAF-1 OFF, and HOXA9 OFF iGMPs. **f,** Experimental schematic illustrating concomitant growth arrest with either uninduced, CAF-1 OFF, or HOXA9 OFF cells (see methods). In CAF-1 OFF condition, cells are pretreated with IPTG for 12 h to achieve Chaf1b knockdown at the time of growth arrest induction. **g,** Cell cycle profile of SCF titrations measured by DNA content (DAPI) after 12 h of SCF withdrawal in uninduced and CAF-1 OFF cells. 1x SCF reflects normal self-renewing growth conditions and 0.1x or 0.02x SCF reflect SCF withdrawal. **h,** Representative flow cytometry histogram of GFP signal in corresponding SCF concentrations in all conditions. In uninduced iGMPs, cells were analyzed 60 h post growth arrest. In CAF-1 OFF and HOXA9 OFF conditions, cells were analyzed at 48 and 72 h post induction, respectively. **i**, Mean and standard deviation of percent GFP positive populations for all conditions cultured in different SCF concentrations. Data was collected from n=3 independent experiments. Two-way ANOVA with Dunnett's test. *$p<0.0001$. Source data are provided as a Source Data file.

Supplementary Figure 4

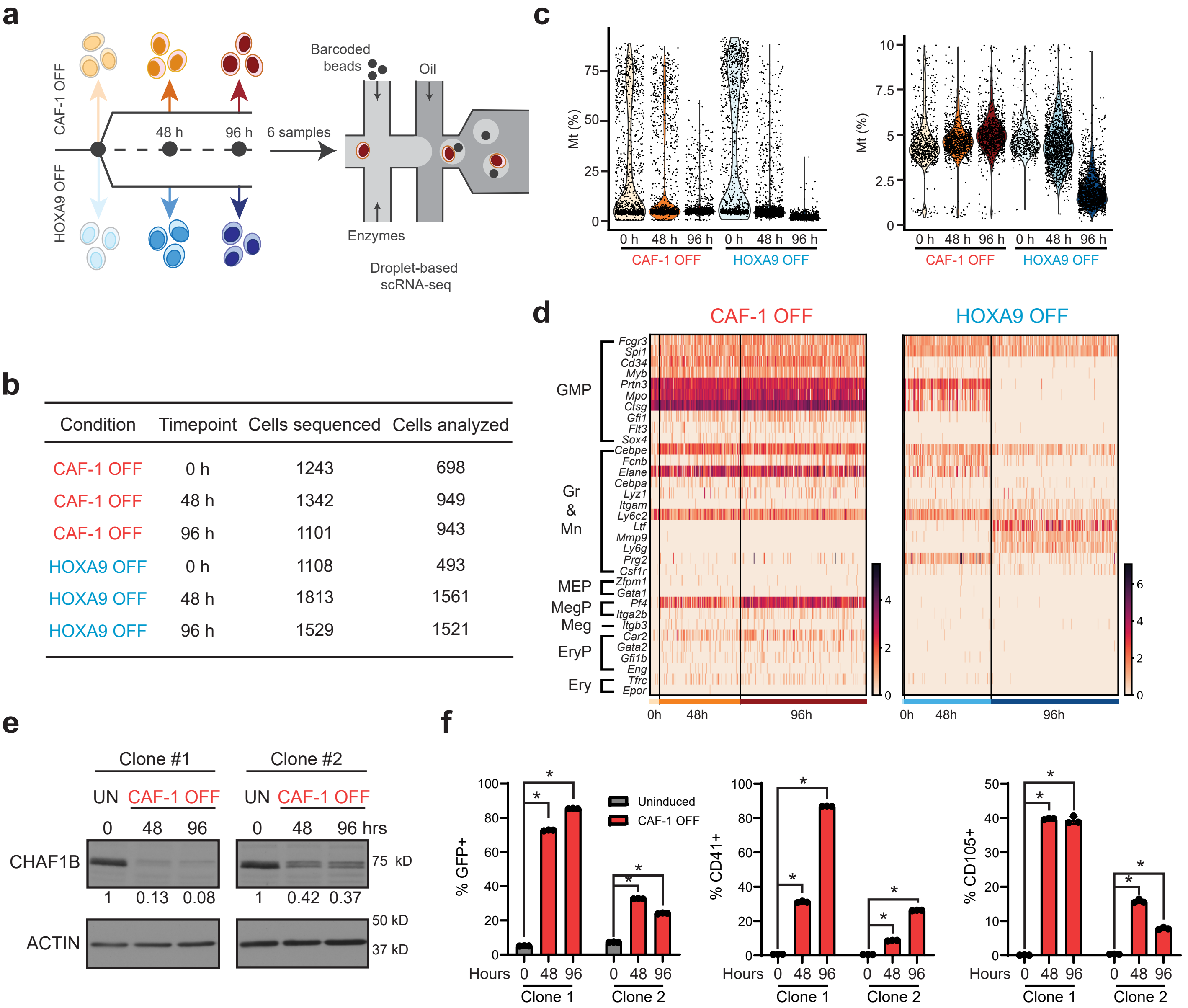

Condition	Timepoint	Cells sequenced	Cells analyzed
CAF-1 OFF	0 h	1243	698
CAF-1 OFF	48 h	1342	949
CAF-1 OFF	96 h	1101	943
HOXA9 OFF	0 h	1108	493
HOXA9 OFF	48 h	1813	1561
HOXA9 OFF	96 h	1529	1521

Supplementary Figure 4. CAF-1 depletion in iGMPs induces a mixed lineage state (Related to Figure 3)

a, Experimental design for scRNA-seq of uninduced, HOXA9 OFF, and CAF-1 OFF conditions depicting the time course analysis at 0, 48, and 96 hours. **b**, Summary of the number of single cells sequenced and analyzed after passing all the quality controls in each condition and time point. **c**, Percentage of mitochondrial UMIs per cell per sample before (left) and after (right) filtering (see methods). **d**, Heatmap of Gr1 expressing cells in CAF-1 OFF and HOXA9 OFF conditions displaying gene expression patterns for a curated set of lineage specific genes (surface markers and transcription factors) as shown in Fig. 3d for all single cells without filtering by Gr1 expression. GMP, granulocyte-macrophage progenitor; Gr, granulocyte; Mn, monocyte/macrophage; MEP, megakaryocyte-erythroid progenitor; MegP, megakaryocyte progenitor; Meg, megakaryocyte, EryP, erythroid progenitor; Ery, erythrocyte. **e**, Western blot analysis of CHAF1B knockdown in IPTG-inducible shChaf1b subclones with variable knockdown levels. Clone #2 having weaker knockdown compared to Clone #1. **f**, Time course flow cytometric analysis of GFP, CD41, and CD105 activation in both clones showing a dosage dependent phenotypic effect. Plotted is the mean and standard deviation of percent positive marker populations in uninduced and CAF-1 OFF iGMPs from n=3 independent experiments. Two-way ANOVA with Dunnett's test (*p<0.0001). Source data are provided as a Source Data file.

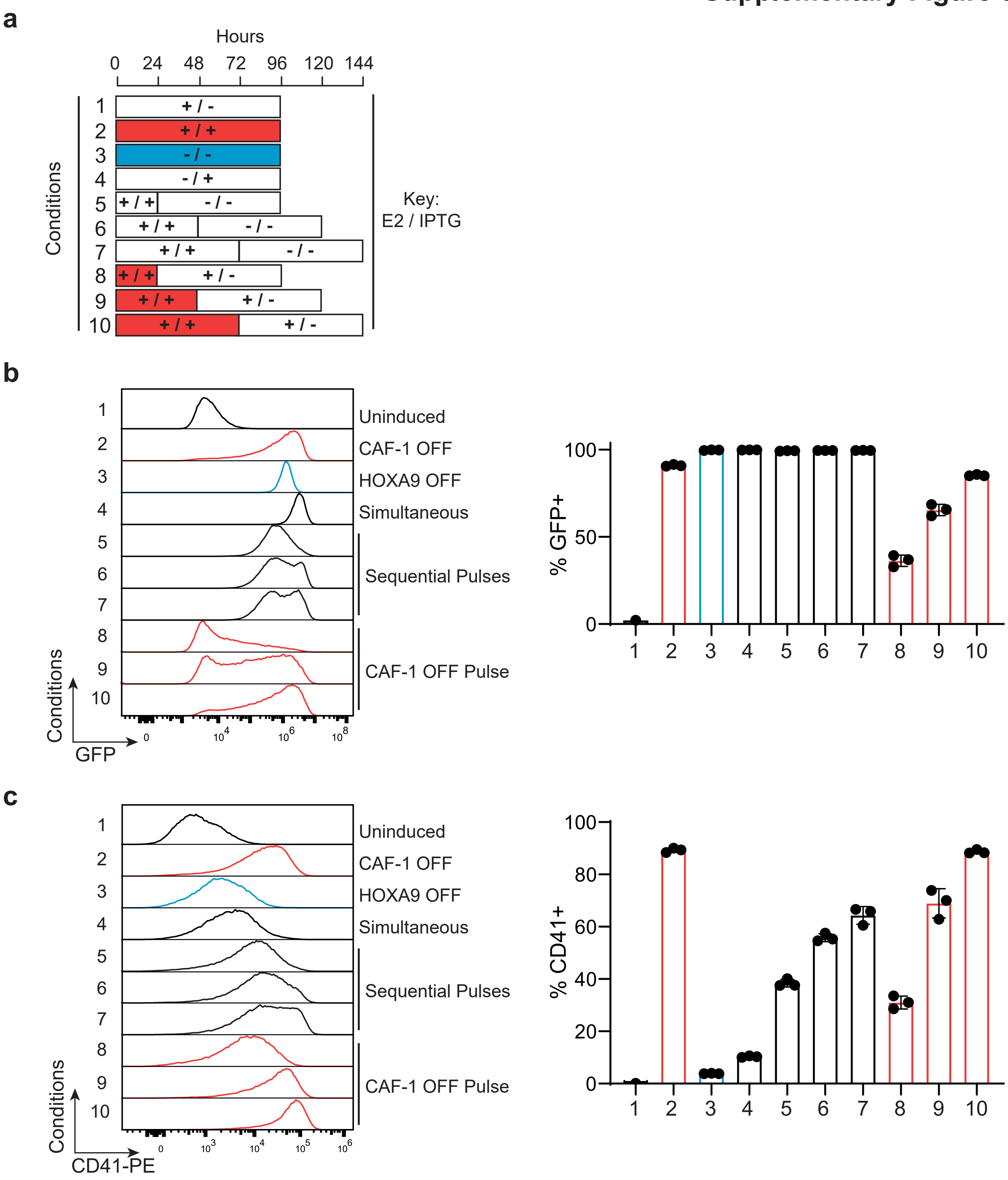

Supplementary Figure 5. The mixed lineage state of iGMPs upon CAF-1 depletion does not depend on HOXA9 activity (Related to Figure 3)

a, Experimental strategy showing uninduced cells and individual CAF-1 OFF or HOXA9 treatments (#1-3), a simultaneous treatment of CAF-1 OFF and HOXA9 OFF (#4), and incremental pulses of CAF-1 OFF followed sequentially by continuous HOXA9 OFF treatment as in Fig. 3h (#5-7) or a pulse+chase period as in Fig. 2e (#8-10). See also Fig. 2e and Fig. 3h. **b-c**, Flow cytometric analysis of GFP and CD41 activation in all conditions shown **(a)**. Left: representative histograms showing the signal for each marker in all conditions. Right: Mean and standard deviation of percent marker positive population for each condition collected from n=3 independent experiments. Source data are provided as a Source Data file.

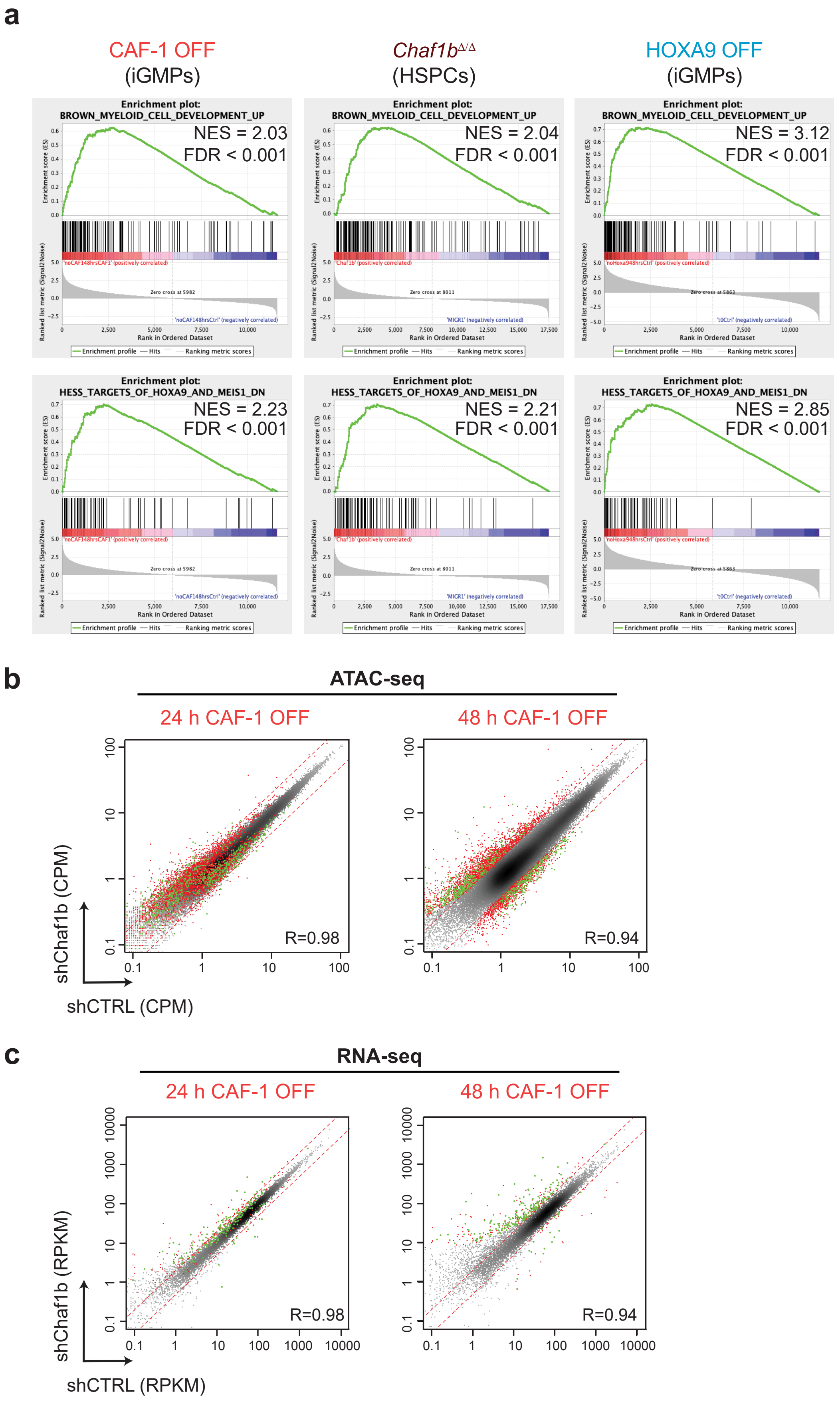

Supplementary Figure 6. Profiling of CAF-1 depletion in iGMPs and primary hematopoietic stem and progenitor cells (Related to Figures 4 and 5)

a, Gene set enrichment analysis (GSEA) of RNA-seq at 48 h post induction of CAF-1 OFF or HOXA9 OFF in iGMPs and *Chaf1b* deletion (*Chaf1b*$^{\Delta/\Delta}$) in primary HSPCs. Calculated normalized enrichment scores (NES) is shown for two significantly enriched gene sets;

myelopoiesis (BROWN_MYELOID_CELL_DEVELOPMENT_UP) and genes downregulated in hematopoietic precursor cells conditionally expressing HOXA9 and MEIS1 (HESS_TARGETS_OF_HOXA9_AND_MEIS1_DN). **b,** Correlation analysis of global ATAC-seq signal (CPM, counts per million mapped reads) comparing uninduced iGMPs to CAF-1 OFF iGMPs at 24 and 48 hours. Data points outside the dotted lines are differentially accessible regions (DARs) with fold change > 2 and FDR < 0.01 cut offs. Red and green colored dots represent unique and common DARs between CAF-1 OFF and HOXA9 OFF from the 48 h time point, respectively (see Fig. 5b). **c**, Correlation analysis of gene expression (RPKM, reads per kilobase of transcript per million mapped reads) comparing uninduced iGMPs to CAF-1 OFF iGMPs at 24 and 48 hours. Genes outside the dotted lines are differentially expressed genes (DEGs) with fold change > 2 and FDR < 0.01. Red and green colored dots represent unique and common DEGs between CAF-1 OFF and HOXA9 OFF from the 48 h time point, respectively (see Fig. 4f).

Supplementary Figure 7

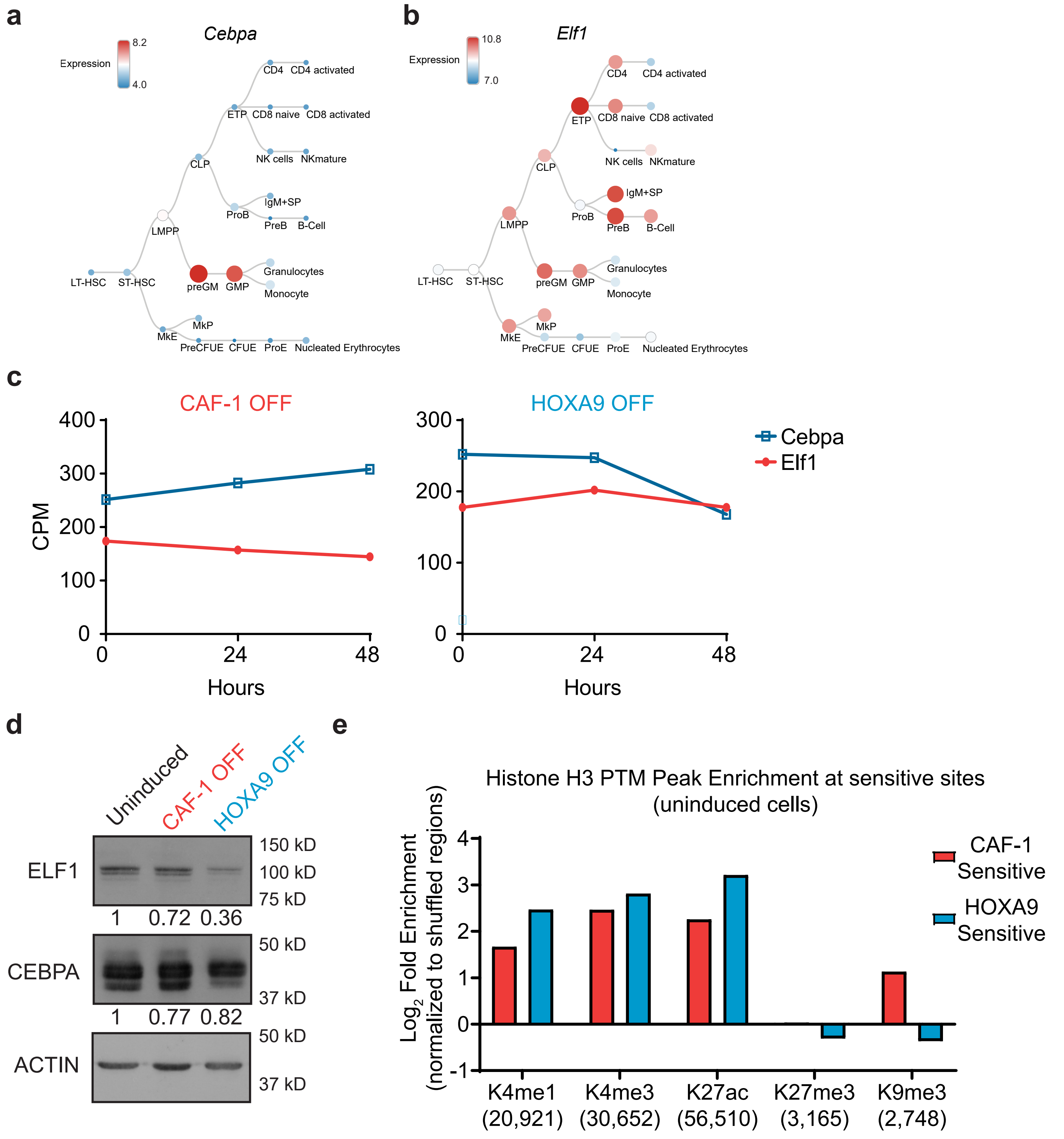

Supplementary Figure 7. TF expression dynamics and characterization of CAF-1 sensitive sites (Related to Figure 5)

a, b, *Cebpa* and *Elf1* expression profile in hematopoietic hierarchical trees from blood spot database using the mouse normal hematopoietic system dataset. **c,** RNA-seq analysis showing expression profiles (CPM, counts per million) of transcription factors *Cebpa* and *Elf1* in uninduced iGMPs (0 hours) and in either CAF-1 OFF or HOXA9 OFF 24 and 48 hours post induction. **d,** Protein levels of ELF1 and CEBPA in uninduced iGMPs and in either CAF-1 OFF or HOXA9 OFF 48 hours post induction. Two independent experiments were performed with similar results but only one representative is shown. **e**, Peak enrichment of histone H3 post translational modifications (PTMs) in uninduced iGMPs that overlap with either CAF-1 sensitive or HOXA9 sensitive sites compared to shuffled genomic regions. Plotted is the Log_2 fold enrichment of called peaks for H3 PTMs at open DARs (see methods). Fraction of overlapped peaks is normalized by the fraction of peaks

overlapping shuffled regions to calculate the fold enrichment of peaks. All positive enrichment of histone marks is statistically significant by χ^2 test ($p < 5x10^{-9}$). Total number of peaks called for each histone mark are labeled under the histone marks. Source data are provided as a Source Data file.

Supplementary Figure 8

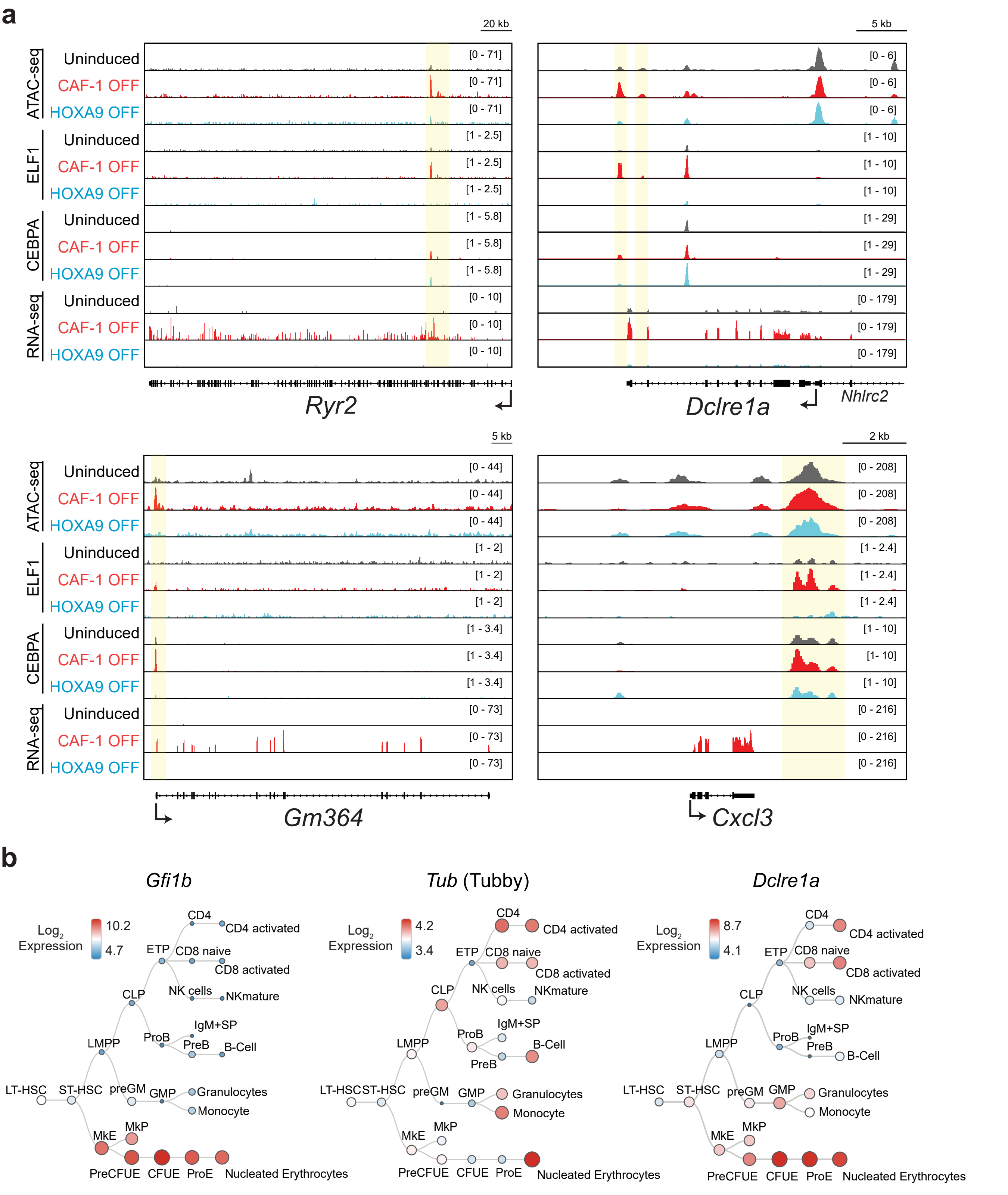

Supplementary Figure 8. CAF-1 restricts chromatin accessibility of ELF1 to sensitive sites proximal to alternate lineage genes (Related to Figure 5)

a, Representative loci harboring sensitive sites that are proximal to genes upregulated upon CAF-1 depletion. ATAC-seq, TF ChIP-seq and RNA-seq tracks are shown for all conditions: uninduced iGMPs and 48 h post CAF-1 OFF and HOXA9 OFF induction. ATAC-seq and RNA-seq signal is normalized to library size and ChIP-seq signal is normalized to input. Shaded regions highlight sensitive sites with corresponding binding of TFs. **b,** *Gfi1b*, *Tub* (Tubby), and *Dclre1a* expression profiles in hematopoietic hierarchical trees from the blood spot database using the mouse normal hematopoietic system dataset (See Fig. 5k for additional genome snapshots).

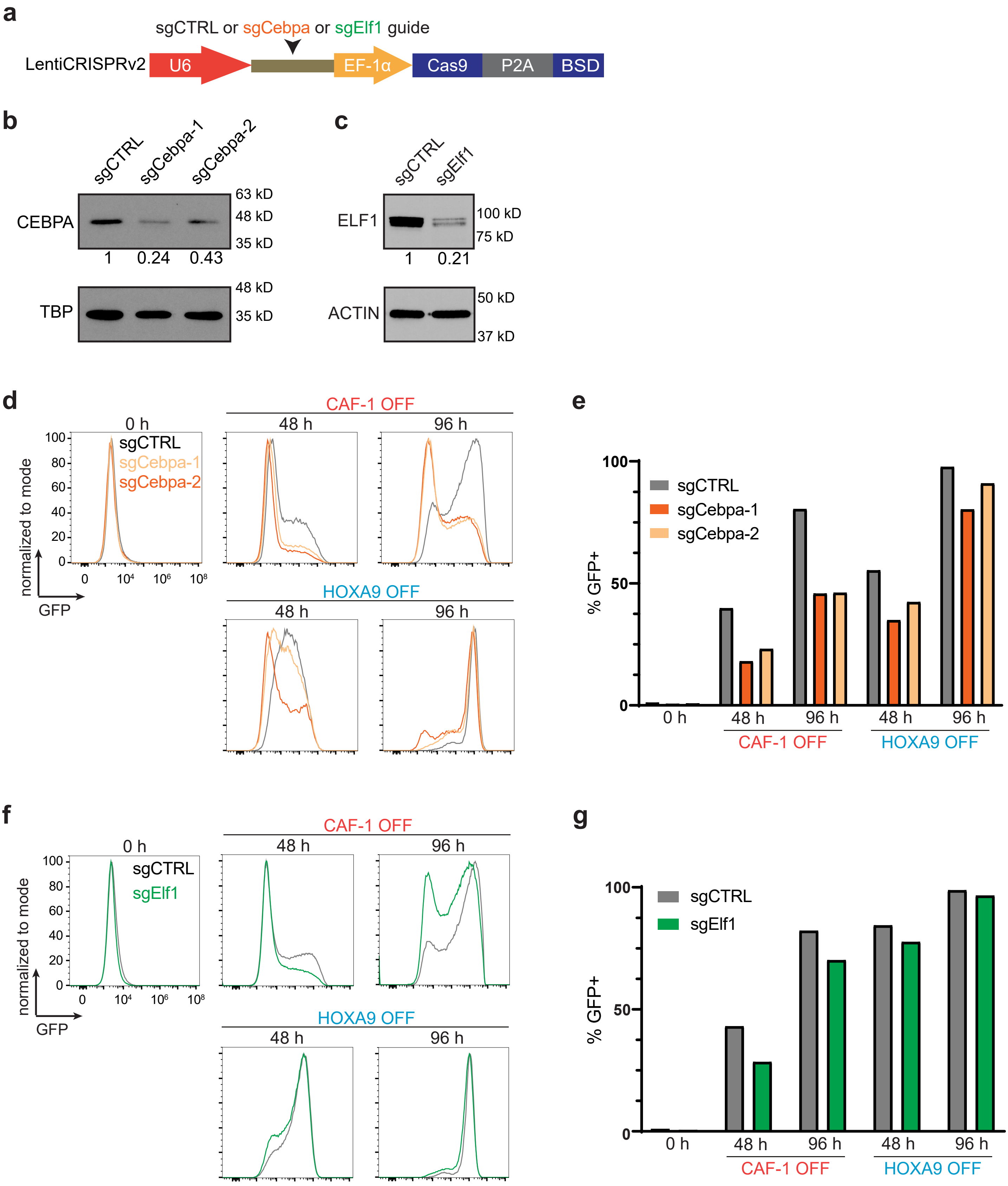

Supplementary Figure 9: CRISPR-CAS9 validation of CEBPA and ELF1 loss of function phenotypes (Related to Figure 6)

a, Schematic of lentiviral CRISPR-CAS9 expression vector (LentiCRISPRv2) co-expressing CAS9 and corresponding single guide RNA (sgRNA) cloned inserts. Cells were transduced with a control vector (sgCTRL) or single guides targeting either *Cebpa* or *Elf1*. U6, U6 promoter; EF-1α, EF-1α promoter; P2A, peptide 2A.

b-g, Myeloid differentiation repression in iGMPs by CRISPR targeting of *Cebpa* and *Elf1*. **b-c,** Western blot analysis of CEBPA **(b)** and ELF1 **(c)** upon CRISPR targeting in polyclonal populations. **d-g,** Flow cytometric time course analysis of Lys-GFP expression upon *Cebpa* **(d-e)** and *Elf1* **(f-g)** targeting in CAF-1 OFF and HOXA9 OFF conditions. Quantifications of percent GFP positive populations from **(d, f)** are shown in **(e, g)**. Two independent experiments were performed with similar results but only one representative is shown. See Fig. 6 for additional confirmation of the phenotype with shRNA mediated knockdown of CEBPA and ELF1. Source data are provided as a Source Data file.

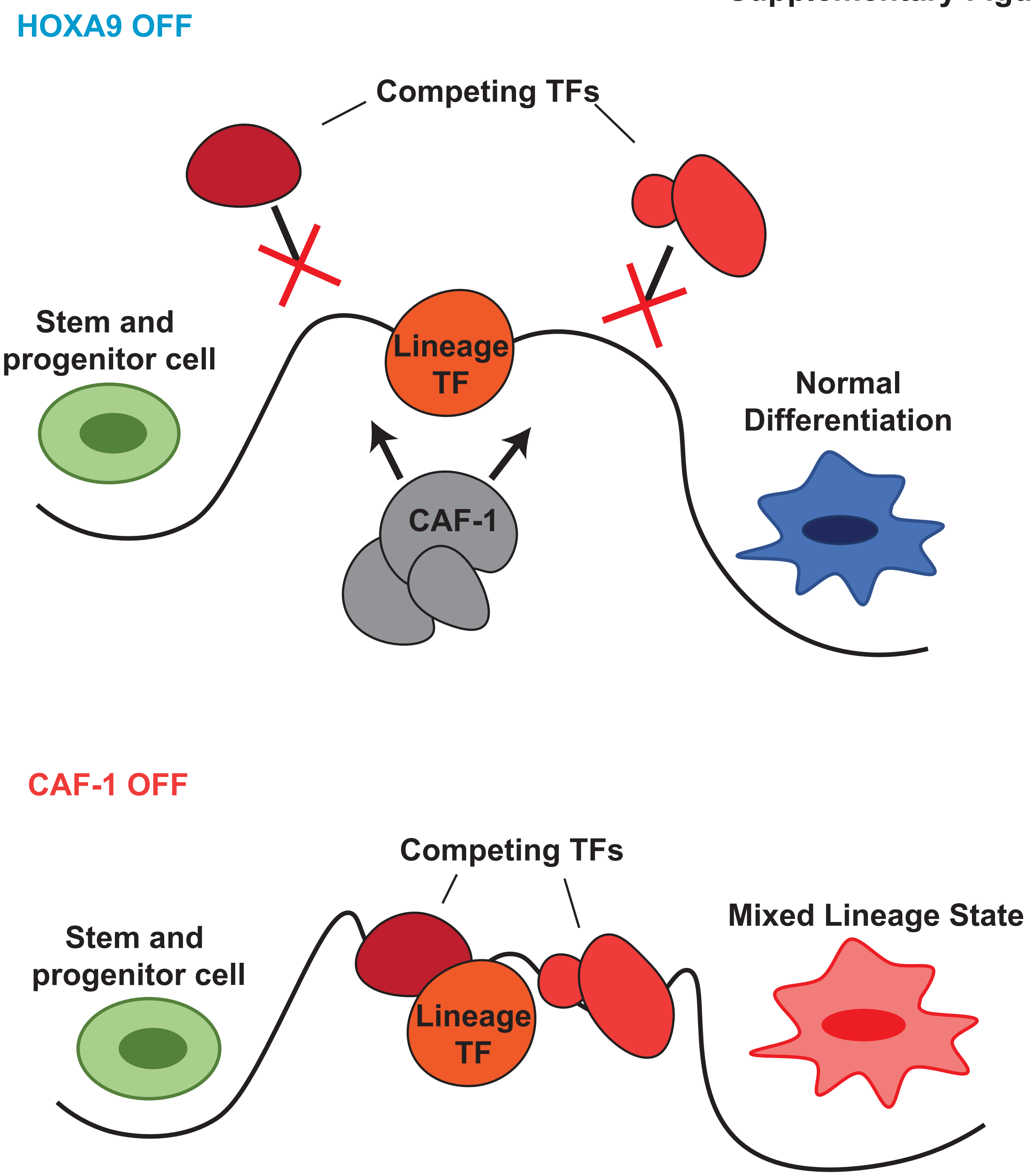

Supplementary Figure 10: Model of altered differentiation path upon chromatin accessibility manipulation via CAF-1 suppression.

During normal stem and progenitor cell differentiation, the control of chromatin accessibility by CAF-1 allows proper binding of lineage-specific transcription factors to their canonical sites, preventing their ectopic binding to alternate sites. CAF-1 depletion promotes chromatin opening at specific loci, leading to competitive binding of transcription factors to these sites and driving alternative lineage genes, which leads to a mixed cellular state.

Supplementary Table 1. List of oligonucleotide sequences

Type	Name	Sequence
qPCR Primer	β-actin-F	CGCCACCAGTTCGCCATGGA
qPCR Primer	β-actin-R	TACAGCCCGGGGAGCATCGT
qPCR Primer	Cebpa-F	ATAGACATCAGCGCCTACATCGA
qPCR Primer	Cebpa-R	GTCGGCTGTGCTGGAAGAG
qPCR Primer	Chaf1a-F	GTGTCTTCCTCAACTTTCTCCTTGG
qPCR Primer	Chaf1a-R	CCGCGGCCGTGGATTGC
qPCR Primer	Chaf1b-F	CCGCCGTCAGGATCTGGAAGTTG
qPCR Primer	Chaf1b-R	GCTCCTTGCTGTCATTCATCTTCCAC
qPCR Primer	Cxcl3-F	GAAAGGAGGAAGCCCCTCAC
qPCR Primer	Cxcl3-R	ACACATCCAGACACCGTTGG
qPCR Primer	Elf1-F	TGCAAGTAACGGCATGGAGG
qPCR Primer	Elf1-R	AGGAACATGTTCCACAATAACAGCA
qPCR Primer	Gapdh-F	CATGGCCTTCCGTGTTCCTA
qPCR Primer	Gapdh-R	GCCTGCTTCACCACCTTCTT
qPCR Primer	Gfi1b-F	CAGTCCTACGCCCACCTTGG
qPCR Primer	Gfi1b-R	AGGGTGGATGAACGCTTGAA
qPCR Primer	Tub(Tubby)-F	AGGCAGCAGAAGCTCGAC
qPCR Primer	Tub(Tubby)-R	TCGGCCTCTTGAACTTGGTAG
shRNA	shChaf1b-1 (TRCN0000318223)	GCTGTCAATGTTGTACGCTTT
shRNA	shChaf1b-2 (TRCN0000318224)	CGTCATTCTGTTGTGGAAGAT
shRNA	shCebpa-1	GCCGAGATAAAGCCAAACAAC
shRNA	shCebpa-2	GGACAAGAACAGCAACGAGTA
shRNA	shElf1	GTGATCCTGCTATATTTCCTG
shRNA	shCTRL (scrambled shRNA)	CCTAAGGTTAAGTCGCCCTCGC
shRNA	shCTRL (luciferase targeting)	CAACAAGATGAAGAGCACCAA
sgRNA	sgCebpa-1	AGAAGTCGGCCGACTCCATG
sgRNA	sgCebpa-2	GCGGCGCGGTCATGTCCGCG
sgRNA	sgElf1	ATGAACAGTTCGGAAGAGCT
