# Supplementary material for: Regulation of chromatin accessibility by the histone chaperone CAF-1 sustains lineage fidelity
Source: Nat Commun. 2022 Apr 29;13:2350. doi: 10.1038/s41467-022-29730-6 (PMC9054786; doi:10.1038/s41467-022-29730-6)
Supplement: Supplementary file 2 — Description of Additional Supplementary Files [file 41467_2022_29730_MOESM2_ESM.docx]

**Description of Additional Supplementary Files**

**Supplementary Data 1:** Transcriptional profile of CAF-1 OFF iGMPs and lineage annotation of upregulated genes upon CAF-1 depletion. Sheet1: Gene expression changes in CAF-1 OFF iGMPs after 48 hours of induction compared to uninduced cells. Columns provide the following information: gene ID (A) , Log2 fold changes (FC; B) and false discovery rates values (C). Sheet2: Genes that are commonly upregulated between CAF-1 OFF iGMPs and Chaf1b-deleted HSPCs only (n= 42; see Fig. 4f) are listed and annotated based on their expression in hematopoietic lineages using the mouse normal hematopoietic system dataset in the BloodSpot database. Columns provide the following information Gene ID (A) and lineage annotation (B) followed by values of FC and FDR for each corresponding condition and cell type.
