# Supplementary material for: Regulation of chromatin accessibility by the histone chaperone CAF-1 sustains lineage fidelity
Source: Nat Commun. 2022 Apr 29;13:2350. doi: 10.1038/s41467-022-29730-6 (PMC9054786; doi:10.1038/s41467-022-29730-6)
Supplement: Supplementary file 4 — Reporting Summary [file 41467_2022_29730_MOESM4_ESM.pdf]

nature research

Corresponding author(s): Sihem Cheloufi, Andrew Volk, and Jernej Murn

Last updated by author(s): Feb 19, 2022

Reporting Summary

Nature Research wishes to improve the reproducibility of the work that we publish. This form provides structure for consistency and transparency in reporting. For further information on Nature Research policies, see our Editorial Policies and the Editorial Policy Checklist.

Statistics

For all statistical analyses, confirm that the following items are present in the figure legend, table legend, main text, or Methods section.

n/a	Confirmed	
☐	☒	The exact sample size (*n*) for each experimental group/condition, given as a discrete number and unit of measurement
☐	☒	A statement on whether measurements were taken from distinct samples or whether the same sample was measured repeatedly
☐	☒	The statistical test(s) used AND whether they are one- or two-sided<br>*Only common tests should be described solely by name; describe more complex techniques in the Methods section.*
☒	☐	A description of all covariates tested
☐	☒	A description of any assumptions or corrections, such as tests of normality and adjustment for multiple comparisons
☐	☒	A full description of the statistical parameters including central tendency (e.g. means) or other basic estimates (e.g. regression coefficient) AND variation (e.g. standard deviation) or associated estimates of uncertainty (e.g. confidence intervals)
☐	☒	For null hypothesis testing, the test statistic (e.g. F, t, r) with confidence intervals, effect sizes, degrees of freedom and P value noted<br>*Give P values as exact values whenever suitable.*
☒	☐	For Bayesian analysis, information on the choice of priors and Markov chain Monte Carlo settings
☒	☐	For hierarchical and complex designs, identification of the appropriate level for tests and full reporting of outcomes
☐	☒	Estimates of effect sizes (e.g. Cohen's d, Pearson's r), indicating how they were calculated

Our web collection on statistics for biologists contains articles on many of the points above.

Software and code

Policy information about availability of computer code

Data collection	Flow data was collected with NovoExpress (v1.5) and FACSDiva software (v9.0).<br>RT-qPCR data was collected with CFX Maestro (v2.2).<br>Sequencing data was collected by Illumina NVCS/RTA software (v1.7/v3.4.4).<br>Luminescence data was collected with GloMax-Multi Detection System External PC Connect Kit (v1.1.14, Cat.# E8916).<br>Morphological images were acquired with Nikon Elements (v5.11.01)
Data analysis	Statistical analysis and graph generation was conducted with GraphPad Prism (v8.0, v9.2) and R (v3.6).<br>Flow cytometry data was analyzed with FlowJo (v9, v10).<br>Immunoblots were analyzed with ImageJ (v1.53c).<br><br>Sequencing analysis software includes:<br>Read Alignment: Bowtie2 (v2.2.9, v2.4.1), BWA (v0.7.17), STAR (v2.7).<br>Read/alignment processing: cutadapt (v2.1), bamUtil (v1.0.14), Picard MarkDuplicates (v2.23.3).<br>Peak calling: SEACR (v1.3), MACS2 (v2.2.6).<br>scRNA-seq Analysis: Seurat (v3.0.1), CellRanger (v3.1.0).<br>Differential analysis: edgeR (v3.14).<br>Downstream analysis: DeepTools (v3.5.0, v3.3.0; bamCoverage, computeMatrix), bedmap (v2.4.38), bedtools (v2.29.2), IGV (v2.9.4), GSEA (v4.2).

For manuscripts utilizing custom algorithms or software that are central to the research but not yet described in published literature, software must be made available to editors and reviewers. We strongly encourage code deposition in a community repository (e.g. GitHub). See the Nature Research guidelines for submitting code & software for further information.

Data

Policy information about availability of data

All manuscripts must include a data availability statement. This statement should provide the following information, where applicable:

- Accession codes, unique identifiers, or web links for publicly available datasets
- A list of figures that have associated raw data
- A description of any restrictions on data availability

The scRNA-seq, RNA-seq, ATAC-seq, CUT&RUN and ChIP-seq data generated in this study have been deposited in the Gene Expression Omnibus (GEO) database and are publicly available under accession number GSE158229 (https://www.ncbi.nlm.nih.gov/geo/query/acc.cgi?acc=GSE158229). Genome assemblies used in this paper (GRCm38/mm10, GCF_000001635.2 and MGSCv37/mm9, GCF_000001635.18) are publicly available. Uncropped immunoblots and source data underlying graphs are presented in the "Source Data" file for Figures 1, 2, 4, 5, and 6, and Supplementary Figures 1, 3, 4, 5, 7, and 9. Additional RNA-seq data supporting the findings of this study are presented in a Supplementary Data file. The reporting summary for this article is available under supplementary information. Source data are provided with this paper.

Field-specific reporting

Please select the one below that is the best fit for your research. If you are not sure, read the appropriate sections before making your selection.

☒ Life sciences ☐ Behavioural & social sciences ☐ Ecological, evolutionary & environmental sciences

For a reference copy of the document with all sections, see nature.com/documents/nr-reporting-summary-flat.pdf

Life sciences study design

All studies must disclose on these points even when the disclosure is negative.

Sample size	Sample sizes were not predetermined based on statistical methods, but were chosen according to the standards for molecular and phenotypic characterization of in vivo and primary cellular models. At least three independent replicates for each condition were performed. For experiments with n < 3, results were validated in independent clones, independent experiments, additional systems, and/or with orthogonal methods as described in the article.
Data exclusions	Data were only excluded for failed experiments resulting from technical issues.
Replication	Experiments were repeated in triplicates to confirm experimental reproducibility. Multiple biological samples were tested to confirm biological reproducibility. All results reported in the manuscript have been reproduced in independent experiments.
Randomization	Cells and animals were randomly allocated into experimental groups.
Blinding	Blinding was not utilized due to objective data collection methods using quantitative measurements that did not require subjective interpretation or judgment.

Reporting for specific materials, systems and methods

We require information from authors about some types of materials, experimental systems and methods used in many studies. Here, indicate whether each material, system or method listed is relevant to your study. If you are not sure if a list item applies to your research, read the appropriate section before selecting a response.

Materials & experimental systems

n/a	Involved in the study	
☐	☒	Antibodies
☐	☒	Eukaryotic cell lines
☒	☐	Palaeontology and archaeology
☐	☒	Animals and other organisms
☒	☐	Human research participants
☒	☐	Clinical data
☒	☐	Dual use research of concern

Methods

n/a	Involved in the study	
☐	☒	ChIP-seq
☐	☒	Flow cytometry
☒	☐	MRI-based neuroimaging

Antibodies

Antibodies used	CEBPA (CST, Clone D56F10, Cat#s: 8178S [1:1,000 WB], 8178BF [10 µg ChIP])<br>ELF1 (Bethyl, Cat#: A301-443A [10 µg ChIP])<br>H3K27ac (Abcam, Cat#: ab4729 [2 µg ChIP])<br>H3K27me3 (CST, Clone C36B11, Cat#: 9733 [1:100 C&R])

April 2020

H3K4me3 (Abcam, Cat#: ab8580 [2 µg ChIP])
H3K9me3 (Abcam, Cat#: ab8898 [1:100 C&R])
H3K4me1 (Abcam, Cat#: ab8895 [1:100 C&R])
IgG (Cell Signaling, Cat#: 2729S [1:100 C&R])
CD11b (Biolegend, Clone M1/70, Cat#: 101216 [1:200 FC])
Gr1 (Biolegend, Clone RB6-8C5, Cat#: 108407 [1:200 FC])
CD41 (Biolegend, Clone MWReg30, Cat#: 133905 [1:200 FC])
CD105 (Biolegend, Clone MJ7/18, Cat#: 120407 [1:200 FC])
CD11b (eBioscience, Clone M1/70, Cat#: 12-0112-82 [1:200 FC])
CD117 (eBioscience, Clone 2B8, Cat#: 17-1171-82 [1:200 FC])
Gr1 (eBioscience, Clone RB6-8C5, 17-5931-82 [1:200 FC])
Chaf1a (SCBT, Clone D-16, Cat#: sc10206 [1:1,000 WB])
Chaf1b (SCBT Clone B-10, Cat#: sc393662 [1:1,000 WB])
ELF1 (SCBT, Clone C-4, Cat#: sc133096 [1:1,000 WB])
ER (CST, CloneD8H8, Cat#: 8644T [1:1,000 WB])
HOXA9 (Novus Bio., Cat#: NBP2-32356, [1:1,000 WB])
TBP (Abcam, Clone 1TBP18, Cat#: ab818 [1:1,000 WB])
alpha-Tubulin (CST, Cat#: 2144S [1:1,000 WB])
beta-ACTIN-HRP (Millipore Sigma, Clone AC-15, Cat#: A3854 [1:20,000 WB])
anti-Rabbit-HRP (Secondary Ab, Millipore Sigma, Cat#: AP124P [1:2,000 WB])
anti-Mouse-HRP (Secondary Ab, Millipore Sigma, Cat#: AP307P [1:2,000 WB])

Validation

We validated CEBPA (CST, Clone D56F10) and ELF1 (Bethyl, Cat#: A301-443A) antibodies by western blots and intracellular FACS using knockdown cells for each TF. In addition, we validated specificity of ChIP-seq data using motif prediction of target peaks.
CEBPA (CST, Clone D56F10, Cat#s: 8178S, 8178BF): manufacturer validated for IP and WB with mouse samples. Validated in literature for ChIP-seq in mouse samples (PMID: 30423293).
ELF1 (Bethyl, Cat#: A301-443A): manufacturer validated for WB with human samples and validated by ENCODE for ChIP-seq.
H3K27ac (Abcam, Cat#: ab4729): manufacturer validated for ChIP-seq with mouse samples and in numerous publications.
H3K27me3 (CST, Clone C36B11, Cat#: 9733): manufacturer validated for ChIP-seq with mouse samples and in numerous publications.
H3K4me3 (Abcam, Cat#: ab8580): manufacturer validated for ChIP-seq with mouse samples and in numerous publications.
H3K9me3 (Abcam, Cat#: ab8898): manufacturer validated for ChIP-seq with mouse samples and in numerous publications.
H3K4me1 (Abcam, Cat#: ab8895): manufacturer validated for ChIP-seq with mouse samples and in numerous publications.
IgG (Cell Signaling, Cat#: 2729S): manufacturer validated for ChIP-seq with mouse samples and in numerous publications
pAG-MNase (Epicypher, Cat#: 15-1116): manufacturer validated for CUT&RUN.
CD11b (Biolegend, Clone M1/70, Cat#: 101216): manufacturer validated for flow cytometry with mouse samples and in numerous publications.
Gr1 (Biolegend, Clone RB6-8C5, Cat#: 108407): manufacturer validated for flow cytometry with mouse samples and in numerous publications.
CD41 (Biolegend, Clone MWReg30, Cat#: 133905): manufacturer validated for flow cytometry with mouse samples and in numerous publications.
CD105 (Biolegend, Clone MJ7/18, Cat#: 120407): manufacturer validated for flow cytometry with mouse samples and several publications.
CD11b (eBioscience, Clone M1/70, Cat#: 12-0112-82): manufacturer validated for flow cytometry with mouse samples and several publications.
CD117 (eBioscience, Clone 2B8, Cat#: 17-1171-82): manufacturer validated for flow cytometry with mouse samples and several publications.
Gr1 (eBioscience, Clone RB6-8C5, 17-5931-82): manufacturer validated for flow cytometry with mouse samples and several publications.
Chaf1a (SCBT, Clone D-16, Cat#: sc10206, discontinued): manufacturer validated for WB with mouse samples and in lab with Chaf1a knockdown samples.
Chaf1b (SCBT Clone B-10, Cat#: sc393662): manufacturer validated for WB with mouse samples and in lab with Chaf1b knockdown samples.
ELF1 (SCBT, Clone C-4, Cat#: sc133096): manufacturer validated for WB with mouse samples and in lab with Elf1 knockdown samples.
ER (CST, CloneD8H8, Cat#: 8644T): manufacturer validated for WB with human samples and in numerous publications.
HOXA9 (Novus Bio., Cat#: NBP2-32356): manufacturer validated for WB with mouse samples.
TBP (Abcam, Clone 1TBP18, Cat#: ab818): manufacturer validated for WB with mouse samples and in numerous publications.
beta-ACTIN-HRP (Millipore Sigma, Clone AC-15, Cat#: A3854): manufacturer validated for WB with mouse samples and in numerous publications
alpha-Tubulin (CST, Cat#: 2144S): manufacturer validated for WB with mouse samples and in numerous publications.

Eukaryotic cell lines

Policy information about cell lines

Cell line source(s)

Immortalized GMPs were derived as reported previously (PMID: 27641501).
293T (CRL-3216, ATCC)
Chaf1b-targeted C57Bl/6 ESCs (EUCOMM)
CHO-SCF (gift from David Sykes Lab).

Authentication

Cell lines were authenticated by morphology and determination of differentiation potential.

Mycoplasma contamination

Cell lines routinely tested negative for mycoplasma.

Commonly misidentified lines
(See ICLAC register)

None of cell lines used in this study are listed in the ICLAC register.

Animals and other organisms

Policy information about studies involving animals; ARRIVE guidelines recommended for reporting animal research

Laboratory animals	C57Bl/6 mice were housed in a controlled environment (20±2°C, 12/12 h light/dark cycle) in a barrier-grade animal facility. Experimental mice were co-housed with control mice. Mice were euthanized at 8-12 weeks via inhalation of carbon dioxide in a chamber followed by cervical dislocation was performed. Male and female mice were used for this study. Sub-strains include Flp+ and Mx1-Cre mice in the C57Bl/6 genetic background.
Wild animals	This study did not involve wild animals.
Field-collected samples	This study did not involve field-collected samples.
Ethics oversight	Northwestern University Committee on the Use and Care of Animals approved all mouse studies (IACUC number: IS00006115).

Note that full information on the approval of the study protocol must also be provided in the manuscript.

ChIP-seq

Data deposition

☒ Confirm that both raw and final processed data have been deposited in a public database such as GEO.

☒ Confirm that you have deposited or provided access to graph files (e.g. BED files) for the called peaks.

Data access links
May remain private before publication.

GEO (GSE158229): https://www.ncbi.nlm.nih.gov/geo/query/acc.cgi?acc=GSE158229

Files in database submission

File name format is Treatment-Antibody_Rep#_[Matched Input]

Independently sonicated chromatin is identified with suffix lables "_in#".
E.g., "Untreated-ELF1_Rep1_in1", "Untreated-H3K27ac_Rep1_in1", and "Untreated-CEBPA_Rep1_in1" samples are matched to the input sample, "Untreated-INPUT_in1".

CUT&RUN (_CnR) samples are matched to the IgG control sample.

Untreated-ELF1_Rep1_in1
Untreated-ELF1_Rep2_in2
IPTG-ELF1_Rep1_in4
IPTG-ELF1_Rep2_in5
NoE2-ELF1_Rep1_in7
NoE2-ELF1_Rep2_in8
Untreated-CEBPA_Rep1_in1
Untreated-CEBPA_Rep2_in3
IPTG-CEBPA_Rep1_in4
IPTG-CEBPA_Rep2_in6
NoE2-CEBPA_Rep1_in7
NoE2-CEBPA_Rep2_in9
Untreated-H3K27ac_Rep1_in1
Untreated-H3K4me3_Rep1_in2
Untreated-H3K4me3_Rep2_in3
Untreated-INPUT_in1
Untreated-INPUT_in2
Untreated-INPUT_in3
IPTG-INPUT_in4
IPTG-INPUT_in5
IPTG-INPUT_in6
NoE2-INPUT_in7
NoE2-INPUT_in8
NoE2-INPUT_in9

Untreated-H3K4me1_CnR
Untreated-H3K27me3_CnR
Untreated-H3K9me3_CnR
Untreated-IgG_CnR

Genome browser session
(e.g. UCSC)

No longer applicable.

Methodology

Replicates

Two experimental replicates for all three conditions were conducted for CEBPA, ELF1, H3K4me3 ChIP-seq profiling. Two independent experiments were conducted for H3K4me1, H3K27ace, H3K27me3 and H3K9me3 ChIP-seq and CUT&RUN mapping with similar

results but only one experiment is shown. Replicates had high agreement.

Sequencing depth

All samples were sequenced to at least 30 million PE100 reads and had 90+% reads mapped to mm9.

Antibodies

We validated CEBPA (CST, Clone D56F10) and ELF1 (Bethyl, Cat#: A301-443A) antibodies by western blots and intracellular FACS using knockdown cells for each TF. In addition, we validated specificity of TF ChIP-seq data using motif analysis of called peaks.
CEBPA (CST, Clone D56F10, Cat#s: 8178BF): manufacturer validated for IP and WB with mouse samples. Validated in literature for ChIP-seq in mouse samples (PMID: 30423293).
ELF1 (Bethyl, Cat#: A301-443A): manufacturer validated for WB with human samples and validated by ENCODE for ChIP-seq.
H3K27ac (Abcam, Cat#: ab4729): manufacturer validated for ChIP-seq with mouse samples and in numerous publications.
H3K27me3 (CST, Clone C36B11, Cat#: 9733): manufacturer validated for ChIP-seq with mouse samples and in numerous publications.
H3K4me3 (Abcam, Cat#: ab8580): manufacturer validated for ChIP-seq with mouse samples and in numerous publications.
H3K9me3 (Abcam, Cat#: ab8898): manufacturer validated for ChIP-seq with mouse samples and in numerous publications.
H3K4me1 (Abcam, Cat#: ab8895): manufacturer validated for ChIP-seq with mouse samples and in numerous publications.
IgG (Cell Signaling, Cat#: 2729S): manufacturer validated for ChIP-seq with mouse samples and in numerous publications

Peak calling parameters

Bowtie2 with default parameters (v2.2.9, v2.4.1, mm9.bowtie2.index)
For ChIP-seq analysis peaks were called using MACS2 (v2.2.6, default with "-g mm --nomodel --extsize 200" settings. All treatment samples were called against their respective input files.)
For CUT&RUN analysis peaks were called using SEACR software (v1.3) in relaxed mode with normalization to IgG control.

Data quality

Samples passed ENCODE quality control criteria. Samples had 4,300-85,000+ peaks with 5-fold enrichment at 5% FDR.

Software

Reads were mapped with Bowtie2 (v2.29, v2.4.1) to mm9 and peaks called with MACS2 (v2.2.6). Reads and alignments were processed with cutadapt (v2.1), bamUtil (v1.0.14), and Picard (v2.23.3). Read-count normalized bigwigs were generated using Deeptools bamCoverage (v3.5.0). All ChIP-seq and CUT&RUN analyses including data generation for raincloud plots, heatmaps, metaplots, and quality control were completed with Deeptools (v3.5.0) and R (v3.6).

Flow Cytometry

Plots

Confirm that:

☒ The axis labels state the marker and fluorochrome used (e.g. CD4-FITC).

☒ The axis scales are clearly visible. Include numbers along axes only for bottom left plot of group (a 'group' is an analysis of identical markers).

☒ All plots are contour plots with outliers or pseudocolor plots.

☒ A numerical value for number of cells or percentage (with statistics) is provided.

Methodology

Sample preparation

Samples were collected and live stained with conjugated antibodies for 30 minutes, washed twice in DPBS, and then resuspended in FACS Buffer (5% FBS in DPBS with 1mM EDTA) for flow cytometry analysis.

For cell cycle analysis, cells were incubated with EdU for one hour, washed, fixed, permeabilized, and ClickIT labeled with Azide-AlexaFluor 594. Then, cells were stained overnight with DAPI, washed, resuspended in FACS Buffer, and analyzed by flow cytometry.

Instrument

Agilent Novocyte 2100YB, Novocyte Quanteon, BD Fortessa, and BD LSR2 cytometers.

Software

Flow data was collected with NovoExpress (v1.5.6) and Diva software (v9.0)

Cell population abundance

At least 15,000 cells were analyzed for each flow cytometry assay.

Gating strategy

Cells are gated on the live cell population by forward and side scatter and then on single cells by height vs area scatter. Live cells are gated on the viability dye negative population. Fluorophore signal gating boundaries are determined using negative controls. For each marker, the same gates are applied to the experimental and control samples.

For cell cycle analysis, non-subG1 cells are gated by side scatter and DAPI area and then gated by forward and side scatters. Singles cells are gated with DAPI area vs height. Gated cells are then analyzed for EdU, DAPI, and GFP signals. GFP negative and positive cells are gated for further analysis of EdU and DAPI signals.

☒ Tick this box to confirm that a figure exemplifying the gating strategy is provided in the Supplementary Information.
